# Sensitivity analysis and inverse uncertainty quantification for the left ventricular passive mechanics

**DOI:** 10.1007/s10237-022-01571-8

**Published:** 2022-04-04

**Authors:** Alan Lazarus, David Dalton, Dirk Husmeier, Hao Gao

**Affiliations:** School of Mathematics and Statistics, University of Glasgow, Glasgow, UK

**Keywords:** Holzapfel-Ogden model, Global sensitivity analysis, Inverse-uncertainty quantification, Cardiac model, Gaussian process

## Abstract

Personalized computational cardiac models are considered to be a unique and powerful tool in modern cardiology, integrating the knowledge of physiology, pathology and fundamental laws of mechanics in one framework. They have the potential to improve risk prediction in cardiac patients and assist in the development of new treatments. However, in order to use these models for clinical decision support, it is important that both the impact of model parameter perturbations on the predicted quantities of interest as well as the uncertainty of parameter estimation are properly quantified, where the first task is *a priori* in nature (meaning independent of any specific clinical data), while the second task is carried out *a posteriori* (meaning after specific clinical data have been obtained). The present study addresses these challenges for a widely used constitutive law of passive myocardium (the Holzapfel-Ogden model), using global sensitivity analysis (SA) to address the first challenge, and inverse-uncertainty quantification (I-UQ) for the second challenge. The SA is carried out on a range of different input parameters to a left ventricle (LV) model, making use of computationally efficient Gaussian process (GP) surrogate models in place of the numerical forward simulator. The results of the SA are then used to inform a low-order reparametrization of the constitutive law for passive myocardium under consideration. The quality of this parameterization in the context of an inverse problem having observed noisy experimental data is then quantified with an I-UQ study, which again makes use of GP surrogate models. The I-UQ is carried out in a Bayesian manner using Markov Chain Monte Carlo, which allows for full uncertainty quantification of the material parameter estimates. Our study reveals insights into the relation between SA and I-UQ, elucidates the dependence of parameter sensitivity and estimation uncertainty on external factors, like LV cavity pressure, and sheds new light on cardio-mechanic model formulation, with particular focus on the Holzapfel-Ogden myocardial model.

## Introduction

An important challenge in the development of mathematical models for complex physiological systems is to establish the degree of identifiably of the model parameters. A mathematically rigorous concept is *structural identifiability*, which is an intrinsic feature of the mathematical structure of the dynamical system and the quantities of interest (QoIs) that it is designed to predict. In simple words, a parameter is structurally identifiable if the same values of the QoIs cannot be obtained with different parameter values. To paraphrase this: if we have two scenarios for which the QoIs take on identical values, then this implies that the parameters are the same. For a rigorous mathematical definition, see e.g. Chis et al. (2011) or Villaverde et al. (2016).

While structural identifiability analysis of linear models is well understood, there are only a few approximate methods for testing the structural identifiability of nonlinear models. This includes the Taylor series method (Pohjanpalo 1978), the generating series method (Walter and Lecourtier 1982), the similarity transformation approach (Vajda et al. 1989), the differential algebra based method (Ljung and Glad 1994), the direct test (Denis-Vidal and Joly-Blanchard 2000), and a method based on the implicit function theorem (Bellu et al. 2007). See Chis et al. (2011) for a review and a comparative evaluation on various benchmark problems. Hadjicharalambous et al. (2015) have studied model identifiability in a cardiac model in diastole using several simplified strain energy functions, including a reduced Holzapfel-Ogden model which only linearly depends on *a* and $a_\text {f}$, but a general extension to more complex models remains challenging.

**Fig. 1:**
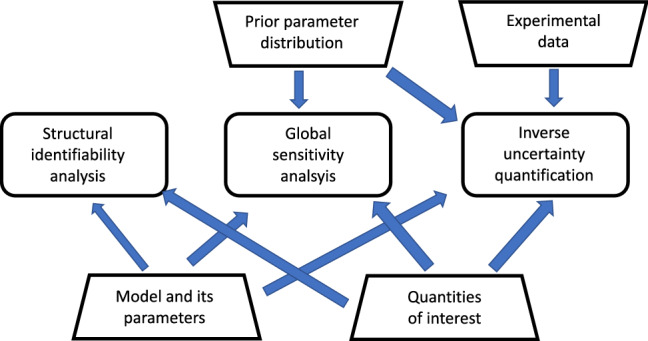
Comparison of *structural identifiability analysis*, *global sensitivity analysis* and *inverse uncertainty quantification*. The structural identifiability of a parameter is an intrinsic feature of the dynamical system and the observation function that defines the QoIs; its global sensitivity additionally depends on the parameters’ prior distribution, and its inverse uncertainty further depends on the experimental data

To avoid the practical challenges of establishing structural identifiability for complex nonlinear systems, *global sensitivity analysis* approaches the question slightly differently and focuses on the contribution of the parameters to the variance of the QoI, either individually (first-order Sobol indices) or in combination with others (total-effects Sobol index). Intuitively, if a parameter contributes a substantial proportion of the variance, the quantity of interest sensitively depends on it. Conversely, a parameter without a significant contribution to the variance has little influence on the quantity of interest. We will review global sensitivity analysis in Sect. 2.2. While structural identifiability is a feature of the dynamical system and the observation function alone, global sensitivity analysis requires the specification of a prior distribution of the parameters. This prior distribution is essential for computing the variance of the quantity of interests. However, the consequence is that global sensitivities are not an intrinsic feature of the dynamical system and the observation function per se, but depend on additional user-defined inputs, as illustrated in Fig. 1.

Structural identifiability is an idealised concept which is based on the assumption that the QoIs can be obtained at infinite precision with zero-noise perturbation. *Practical identifiability*, on the other hand, deals with insufficently informative measurements to determine the parameters with adequate precision. Structural identifiability implies practical identifiability only for an infinite amount of data with zero noise. Wieland et al. (2021) argue that the notion of practical identifiability has been rather vague in the literature, but tends to be related to confidence intervals. In the present article, we treat the concept of structural identifiability equivalent to the concept of *inverse uncertainty quantification.* Inverse uncertainty quantification is about practical identifiability in light of particular experimental data. A parameter that is structurally identifiable is not necessarily practically identifiable if the data are noisy or of insufficient quantity. The additional dependence on experimental data is shown in Fig. 1. We will review the concept of inverse uncertainty quantification in Sect. 2.4.

**Fig. 2:**
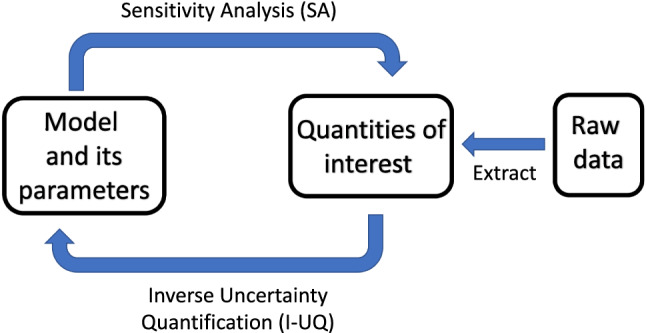
Illustration of sensitivity analysis (SA) and uncertainty quantification (UQ). SA and forward uncertainty quantification (F-UQ) are *a priori* in nature and quantify the impact that perturbations of the model parameters have on the model outputs (the Quantities of Interest: QoIs). Inverse uncertainty quantification (I-UQ) is *a posteriori* in nature and focuses on practical parameter identifiability

The importance of allowing for variability and uncertainty in the computational modelling of complex cardio-physiological systems has been discussed in the seminal white paper by Mirams et al. (2016). In the present article, we quantify variability and uncertainty with sensitivity analysis (SA) and uncertainty quantification (UQ) in the way depicted in Fig. 2. SA is closely related to forward uncertainty quantification (F-UQ), where the aim is to establish how uncertainties in model inputs (such as parameters) affect the model outputs (the QoIs), which is represented by the upper arrow in Fig. 2. This input-output map is an intrinsic feature of the model, and the analysis is *a priori* in nature, meaning that it can be carried out based on the model alone, without need for any measurements or experimental data.

Inverse UQ (I-UQ) focuses on practical identifiability. When parameters are unidentifiable as a result of practical restrictions related to limited availability and quality of data, they are referred to as practically unidentifiable. I-UQ is therefore *a posteriori* in nature, meaning conditionally dependent on the experimental data, and this is represented by the bottom arrow in Fig. 2.

SA can be conceptually divided into local and global SA; see Smith (2014) and Morio (2011) for a review. Local SA merely analyses how small perturbations near an input space value influence the output of the model. Global SA, originally introduced by Sobol (2001), is a more powerful global approach that has been promoted by Saltelli et al. (2010) via the design of computationally efficient quasi-Monte Carlo sampling techniques, and is increasingly being used for the analysis of complex biological systems (Jarrett et al. 2015).

SA techniques have been widely applied in haemodynamic and cardio-vascular modelling. Melis et al. (2017) have carried out global SA for a range of 1D vascular circulation models, following the procedure of Saltelli et al. (2010) and using Gaussian process emulation to reduce the computational complexity. Eck et al. (2016) have compared Monte Carlo and polynomial chaos methods for global SA in 0D and 1D cardiovascular circulation models. Marquis et al. (2017) and Colebank et al. (2019) have combined SA and I-UQ for cardiovascular modelling, by first using SA to select a subset of identifiable parameters, and then using both frequentist and Bayesian I-UQ techniques to obtain confidence and credible intervals of the identifiable parameters. While the first study was restricted to local SA, the second study applied both local and global SA. SA and F-UQ have also been applied to arterial wall mechanics for evaluation of vascular drug therapies (Heusinkveld et al. 2018).

There have been various applications of SA and F-UQ in cardiac electrophysiology (Clayton et al. 2020; Pagani and Manzoni 2021; Mirams et al. 2016), cardiac mechanics (Rodriguez-Cantano et al. 2018; Campos et al. 2019; Kallhovd et al. 2019) and coupled electromechanics (Hurtado et al. 2017; Levrero-Florencio et al. 2020; Rodero et al. 2021). The focus of the present article is on cardiac mechanics, and we will therefore review recent work on SA and UQ in this field in more detail. An overview can be found in Table 1.

**Table 1:** Summary of UQ and SA studies on cardiac mechanics

Studies	Cardiac model	Uncertain inputs	Quantities of interest (QoIs)	UQ and SA	Results
Osnes and Sundnes (2012)	1. Idealized LV 2. Passive filling 3. Fung-type SEF	1. Constitutive parameters 2. Fibre rotation angle	1. LV Cavity volume 2. Apex lengthening & rotation 4. Wall thickness	1. Polynomial chaos expansion 2. Forward UQ	The overall stiffness and cross-fibre stiffness have the greatest influences on QoIs
Rodriguez-Cantano et al. (2018)	1.Iimage-derived LV 2. Passive filling 3. Fung-type SEF	1. Constitutive parameters 2. Random fibre field using a truncated Karhunen-Loeve expansion	1. LV cavity volume 2. Apex lengthening 3. Wall thickness 4. Wall volume	1. Polynomial chaos expansion 2. Sobol sensitivity 3. Forward UQ	The overall stiffness is the most sensitivity parameter; QoIs are relatively insensitive to fibre angle, but sensitive to local variations
Campos et al. (2019)	1. Personalized LV 2. Passive filling 3. Fung-type SEF	1. Constitutive parameters 2. Fibre/sheet angles 3. Wall thickness	1. Wall thickness 2. LV cavity volume 3. The torsion 4. Mean fibre stress/strain	1. Polynomial chaos expansion 2. Forward UQ 3. Sobol sensitivity	Stress is highly sensitivity to wall thickness. Wall thickness has similar proportion of impact on QoIs as material stiffness
Campos et al. (2020)	1. Personalized LV 2. Whole cardiac cycle 3. Fung-type SEF	1. Constitutive parameters 2. Fibre orientations 3. Wall thickness 4. Contractility 5. The circulatory model	1. LV torsion 2. Mean fibre stress/strain 3. Ejection Fraction 4. End-systolic pressure 5. Max pressure variation	1. Polynomial chaos expansion 2. Forward UQ 3. Sobol sensitivity	The most sensitive inputs are wall thickness and contractility
Kallhovd et al. (2019)	1. Image-derived LV 2. Fung-type SEF 3. Passive filling with active contraction	Selected 21 published sets of parameters	1. LV cavity volume 2. Fibre stress/strain 3. Pressure-volume loop	Uncertainty analysis	Similar P-V loops could be obtained by tuning contractility. Local stress is less sensitive to passive parameters, but not strain.
Barbarotta and Bovendeerd (2021)	1. Average LV geometry 2. Fung-type SEF 3. Passive filling with active contraction	Fibre orientation (helix angle and transverse angle) modelled using a 5-parameter rule-based model	End-systolic strain	1. Mean and standard deviation based on the elementary effects method 2. coefficient of variation	Shear strains are more sensitive to fibre orientation than normal strains
Hurtado et al. (2017)	1. Electromechanics model in a bar geometry 2. Isotropic Neo-Hookean material	1. Electrophysiological parameters (conductance, etc) 2. Passive parameters	1. Action potential duration 2. Peak intracellular transient 3. Fibre stretch 4. Active tension	Polynomial chaos expansion	Passive parameters have little impact on the duration of action potential and peak calcium transient
Rodero et al. (2021)	1. Electromechanics model 2. Shape analysis using PCA 3. Fung-type SEF	Geometry variation by adding ± 2/3 SD of each PCA mode to the average mesh	1. Ventricular pressure/volume 2 Peak pressure variation 3. Contraction duration	1. Global sensitivity analysis 2. Forward finite element simulations	PCA modes 2 and 9 are the most important geometrical features in determining LV mechanics
Gao et al. (2015)	1. Personalized LV 2. Passive filling 3. The H-O model	Constitutive parameters	1. Circumferential strains 2. LV cavity volume	Local sensitivity analysis	The isotropic stiffness and the myofibre stiffness are the most sensitive parameters
Levrero-Florencio et al. (2020)	1. Electromechanical model 2. Idealized LV geometry 3. The H-O model	1. Cross-fibre stiffness 2. Contractility 3. Fibre rotation angle 4. Boundary conditions	1. LV ejection fraction 2. Systolic long-axis shortening 3. Systolic wall thickening 4. End-systolic pressure	1. Local sensitivity analysis 2. Global sensitivity analysis using a partial rank correlation coefficient	LV ejection fraction is strongly affected by contractility. The systolic long-axis shortening is sensitive to fibre rotation angle and cross-fibre contraction
This study	1. Image-derived LV 2. Passive filling 3. The H-O model	1. Constitutive parameters 2. Fibre/sheet rotation angels 3. End-diastolic pressure	1. Regional strains 2. LV cavity volume 3. Apex torsion	1. Gaussian process 2. Sobol sensitivity 3. Inverse uncertainty quantification using Markov Chain Monte Carlo	Good identifiability of *a* and $a_{f}$ in the in vivo pressure range; *b* and $b_{f}$ require the inclusion of high pressure data for their reliable inference.

One early local SA study on cardiac mechanics can be found in Geerts et al. (2003), in which the influence of changes in ellipticity, myofibre orientation and material properties on systolic myofibre stress and strain were studied at the left ventricle (LV) equator using idealized LV geometries. Osnes and Sundnes (2012) have investigated F-UQ and global SA for the LV passive filling phase using an idealized geometry and a Fung-type constitutive law, with a focus on replacing intrusive methods (i.e. methods that modify the simulation code) with non-intrusive methods (i.e. methods that do not require changes in the simulation code). The impact of constitutive parameters and fibre orientation uncertainties on ventricular dynamics (LV cavity volume, apex lengthening and rotation, wall thickness) were studied, and they found that the overall stiffness and cross-fibre stiffness had the greatest influences on selected model outputs, while fibre orientation had a minor influence on apex rotation. Rodriguez-Cantano et al. (2018) have extended this work with a focus on the impact of uncertainties in material parameters and fibre orientation field on LV mechanics during diastolic filling. The fibre field variation was approximated using a truncated Karhunen-Loeve expansion, and a polynomial chaos expansion-based method was adopted for F-UQ analysis in a more realistic LV model compared to Osnes and Sundnes (2012). Campos et al. (2019) have extended global SA and F-UQ analysis in LV passive mechanics by considering geometrical uncertainties: wall thickness variation, and they suggested that LV passive mechanics may be more affected by wall thickness than material properties. Later, they (Campos et al. 2020) further extended a similar analysis to the full cardiac cycle by taking into account uncertainties in active stress and the circulatory model. They found that LV ejection fraction and ventricular torsion were very sensitive to active stress, wall thickness and fibre direction, but not the passive material parameters, which was different from the SA and F-UQ in LV passive mechanics. Existing cardiac models usually consider the myofibre transverse angle, which is the angle between the transmural direction and the projected myofibre direction into the transmural-circumferential plane, to be zero. To this end, Barbarotta and Bovendeerd (2021) further studied the sensitivity of systolic strains to myofibre orientation described by the helix and transverse angles. Their results suggested that shear strains are more sensitive to myofibre orientations than normal strains.

The aforementioned studies (Osnes and Sundnes 2012; Rodriguez-Cantano et al. 2018; Campos et al. 2019, 2020) used a transversely isotropic Fung-type constitutive law. Experimental studies have suggested that the myocardium is an orthotropic material with three mutually orthogonal principal axes (Dokos et al. 2002; Holzapfel and Ogden 2009; Sommer et al. 2015), and for that reason, a few orthotropic nonlinear constitutive laws have been proposed, including the widely used H-O model developed by Holzapfel and Ogden (2009).

For the H-O model, Gao et al. (2015) performed local SA for the passive myocardial properties using a patient-specific LV geometry, and they found that the LV passive mechanics are highly sensitive to the isotropic stiffness and myofibre stiffness, and parameters in the H-O model are also highly correlated. Levrero-Florencio et al. (2020) studied local/global SA in an idealized LV electromechanical model with a focus on LV pump function by treating the cross-fibre stiffness as the only uncertain parameter in the H-O model. To the best of our knowledge, a comprehensive global SA and I-UQ analysis using the H-O model has not been reported in the literature despite its wide application in cardiac mechanics (Gao et al. 2017a; Sahli Costabal et al. 2019).

Our work aims to complement previous work on SA and F-UQ in cardio-mechanics models (Osnes and Sundnes 2012; Rodriguez-Cantano et al. 2018; Campos et al. 2020) by including I-UQ with a focus on the H-O model in the LV passive filling. To paraphrase this with reference to Fig. 2: while the previous studies cited above have carried out UQ that is intrinsically *a priori* in nature (F-UQ), corresponding to the top arrow in the diagram, we complement this work with a dimension that is *a posteriori* in nature, corresponding to the bottom arrow in Fig. 2. This enables us to additionally quantify the degree of practical parameter identifiability, in light of experimental data. Our work extends the work of Gao et al. (2015) by carrying out global rather than local SA, and systematically combining it with I-UQ. Our study is conceptually related to the work of Marquis et al. (2017) and Colebank et al. (2019), but shifts the focus from cardiovascular to cardio-mechanics modelling. Besides gaining methodological insights into the relation between forward and inverse uncertainty quantification in the context of the LV passive mechanics, our work leads to a better understanding of the appropriate level of complexity for constitutive cardiac mechanics models, with a particular focus on the H-O strain energy function.

Our paper is structured as follows: we begin with a methodological overview in Sect. 2, covering the LV model (Sect. 2.1), global sensitivity analysis (Sect. 2.2), surrogate modelling and emulation (Sect. 2.3) and posterior inference (Sect. 2.4). This is followed by two sections on our SA and I-UQ work, covering SA in Sect. 3, and I-UQ in Sect. 4. Both sections follow the structure to begin with a description of the experimental setup (Sects. 3.1 and 4.1 ) and the surrogate model training (Sects. 3.2 and 4.2 ), followed by surrogate model validation (Sects. 3.3 and 4.3 ) and a discussion of the results (Sects. 3.4 and 4.4 ). We discuss the relation of our SA and I-UQ results in Sect. 5, and present an outlook on future work in Sect. 6. Finally, Sect. 7 concludes.

## Methods

This section describes the methods used to perform SA and I-UQ for the parameters of a constitutive model for the passive mechanics of the left ventricle. Given that this study involves the application of statistical methods to cardiac mechanics, there will inevitably be an overlap in the standard notation used by the two communities. For this reason, we make explicit our choice of notation for those symbols which may cause confusion in Table 2.

**Table 2:** Nomenclature Table

Symbol	Meaning
$\varvec{\tau }$	Cauchy stress tensor
$\varepsilon _\text {cc}$	Segmental circumferential strains
$\varepsilon _\text {ll}$	Segmental longitudinal strains
$\varepsilon _\text {rr}$	Segmental radial strains
$\sigma ^2$	Variance of Normal distribution
$\sigma _f^2$	Amplitude factor in kernel (15)
*Y*	Model output random variable
*X*	Model input random variable
${\mathbf {X}}$	Vector of model input random variables
${\mathbf {x}}$	Vector of fixed model input values
$\varvec{\xi }$	Noise random variable
${\varvec{\theta }}$	Vector of random parameters in I-UQ

**Fig. 3:**
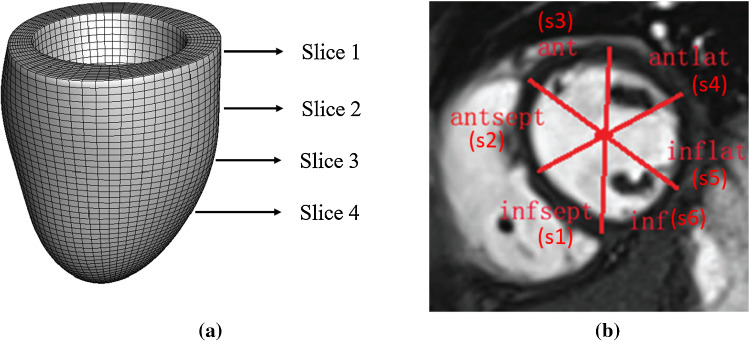
A reconstructed LV geometry with indications of 4 short-axis slices from the base to the mid-ventricle (**a**), and schematic illustration of 6 segmental regions for a selected short-axis slice following the AHA division convention. infsept: inferior septum; antsept:anterior septum; ant: anterior; antlat: anterior lateral; inflat: inferior lateral; inf: inferior

### Left ventricular forward model

#### Patient-specific LV geometry

In this study, a cardiac magnetic resonance (CMR) imaging derived LV model of a healthy volunteer (male, 43 years) is chosen from our previous work (Gao et al. 2017a), which was reconstructed from both the short and long axial CMR cine images at early-diastole following existing studies (Genet et al. 2014). The LV geometry, which is displayed in Fig. 3a, was discretized using hexahedral elements. Further details of LV geometry reconstruction can be found in (Li et al. 2020).

It is common practice to incorporate the layered myofibre structure in the LV models, while due to the extreme difficulty of imaging myofibres in vivo, we adopted a rule based method (RBM) for describing the myofibre structure (Wang et al. 2013), in which a local material coordinate system was defined, the so-called fibre ($\mathbf{m}$)–sheet ($\mathbf{s}$)– sheet-normal ($\mathbf{n}$) system. In the present study, the fibre angle varied linearly from $\alpha _\text {endo}$ at endocardium to $\alpha _\text {epi}$ at epicardium, with the sheet orientation along the transmural direction varying from $45^o$ at endocardium to $-45^o$ at epicardium.

#### Constitutive law of the myocardium

We consider the passive myocardium as an incompressible, anisotropic and hyperelastic material, described by the constitutive law introduced by Holzapfel and Ogden (Holzapfel and Ogden 2009), the so-called H-O model, that is,

1 $$\begin{aligned} {\Psi }&= \frac{a}{2 b}\left[ \exp \left\{ b\left( I_{1}-3\right) \right\} -1\right] \nonumber \\&+\sum _{i \in \{\mathrm {f,s}\}} \frac{a_{i}}{2 b_{i}}\left[ \exp \left\{ b_{i}\left( \max (I_{4 i},1) -1\right) ^{2}\right\} -1\right] \nonumber \\&+\frac{a_{\mathrm {fs}}}{2 b_{\mathrm {fs}}}\left\{ \exp \left( b_{\mathrm {fs}} I_{8 \mathrm {fs}}^{2}\right) -1\right\} \end{aligned}$$

in which *a*, *b*, $a_\text {s}$, $b_\text {s}$, $a_\text {f}$, $b_\text {f}$, $a_\text {fs}$, $b_\text {fs}$ are material constants. In particular, *a* and *b* relate to the isotropic response of the myocardium, while $a_{\mathrm{f}}$ and $b_{\mathrm{f}}$ characterize the reinforced stiffness along the myofibres, the same for $a_\text {s}$ and $b_\text {s}$ along the sheet direction, and finally $a_\text {fs}$, $b_\text {fs}$ describe the shear response between the fiber and sheet directions. The $\max ()$ function in (1) ensures the fibres can only support extension but not compression. The principal invariant $I_1$, the transversely isotropic invariants $I_{\mathrm {4i}}$ and the coupling invariant $I_{8 \mathrm {fs}}$ are calculated from the right Cauchy deformation tensor $\mathbf{C}=\mathbf{F}^{T}\mathbf{F}$ with $\mathbf{F}$ the deformation gradient, and

$$\begin{aligned}&I_{1} ={\text {tr}}({\mathbf{C}}), \quad \quad I_{4 \mathrm {f}} ={\mathbf {m}}_{0} \cdot \left( {\mathbf{C}}{\mathbf {m}}_{0}\right) , \\&I_{4 \mathrm {s}} ={\mathbf {s}}_{0} \cdot \left( {\mathbf{C}}{\mathbf {s}}_{0}\right) , \quad I_{8 \mathrm {fs}} ={\mathbf {m}}_{0} \cdot \left( {\mathbf{C}}{\mathbf {s}}_{0}\right) , \end{aligned}$$

where ${\mathbf {m}}_{0}$ and ${\mathbf {s}}_{0}$ are the unit fibre and sheet directions in the reference configuration.

The H-O model has been used widely in personalized cardiac modelling in recent years (Gao et al. 2017a; Palit et al. 2018; Baillargeon et al. 2014; Sahli Costabal et al. 2019; Guan et al. 2019). Due to the poor identifability of some parameters, different reduced forms have been proposed in the literature, which generally fall into two frameworks. The first of these is to introduce scaling parameters that link multiple parameters together. For example, Palit et al. (2018) used a four dimensional parameterization, *a*, *b*, *Ka*, *Kb* where *Ka* and *Kb* scale the remaining *a* and *b* parameters of the H-O model. Further examples of this type of reparametrization can be found in the literature (Gao et al. 2015; Noè et al. 2019; Davies et al. 2019). The alternative approach is to also ignore several parameters, either by fixing them to constant values or removing them from the model altogether, usually a reduced formula with transverse isotropy. For example, Krishnamurthy et al. (2013) removed the terms depending on $a_{\mathrm{s}}, \,b_{\mathrm{s}}, \,a_{\mathrm{fs}}\text { and } b_{\mathrm{fs}}$ from the model. Hadjicharalambous et al. (2015) set $a_{\mathrm{s}}$ and $a_{\mathrm{fs}}$ equal to 0 and fixed *b* and $b_{\mathrm{f}}$ to constant values, leaving two unknown parameters (*a* and $a_{\mathrm{f}}$). They further showed the bijectivity of the map from this two-dimensional parameter space. However, this bijectivity only holds in the case where end diastolic pressure (EDP) is known and *b* and $b_{\mathrm{f}}$ are fixed. With this in mind, they further proposed learning a ratio of *a* and $a_{\mathrm{f}}$ in the real data case when EDP is not known in advance. This method has been adopted in more recent work (Asner et al. 2016; Hadjicharalambous et al. 2017, 2021). None of the above reparameterizations are based on a comprehensive sensitivity study, but from empirical observations and intuitive insights of myocardial behaviours. To the best of our knowledge, our study is the first to motivate a reparameterization using a proper sensitivity analysis.

#### Simulation of LV passive filling

The LV diastolic filling process is described by a quasi-static pressure-loaded boundary-value problem over the computational domain ($\varOmega$) occupied by the LV geometry.

A linearly ramped pressure from 0 to $\text {EDP}$ mmHg is applied to the endocardial surface, where $\text {EDP}$ is the end-diastolic blood pressure inside the LV cavity. The basal plane is fixed along the longitudinal and circumferential directions, with only expansion in the radial direction allowed. The system of equations at the current configuration ($\varOmega _t$) is given by:

2 $$\begin{aligned} {\left\{ \begin{array}{ll} \nabla \cdot (\varvec{\tau }) = 0 \quad \quad &{}\text {in}\,\, \varOmega _t, \\ \varvec{\tau }\cdot \mathbf{n} = \mathbf{t} &{}\text {on}\,\, \varGamma ^\text {endo}, \\ \text {u}_r = \text {u}_z = 0 &{}\text {on}\,\, \varGamma ^\text {base}, \\ \end{array}\right. } \end{aligned}$$

where $\mathbf{n}$ is the normal direction of the endocardial surface $\varGamma ^\text {endo}$, $\mathbf{t}$ is the traction forced resulted from the loaded pressure at $\varGamma ^\text {endo}$, and $\text {u}_r$ and $\text {u}_z$ are the displacement components along the radial direction and z-axis at the basal surface $\varGamma ^\text {base}$, respectively. Note a cylindrical system is introduced for all nodes in the basal surface, see Fig. 3a. The myocardial Cauchy stress ($\varvec{\tau }$) is derived from the H-O model as

3 $$\begin{aligned} \varvec{\tau }=\mathbf{F}\sum _{i=1,\text {4f},\text {4s},\text {8fs}} \frac{\partial {\Psi } }{\partial I_i} \frac{\partial I_i}{\partial \mathbf{F}} - p{\mathbf {I}}, \end{aligned}$$

in which ${\mathbf {I}}$ is the identity matrix, and *p* is the Lagrange multiplier to enforce the incompressibility.

Equation (2) was solved using the general-purpose finite-element package ABAQUS (Simulia, Providence, RI, USA). Refer to (Wang et al. 2013) for details of the finite-element simulation of LV dynamics in diastole.

#### Input parameters

A total of eleven input parameters to the LV model are considered as random in the SA experiments. They are the eight material parameters from (1): $a, b, a_{\mathrm{f}}, b_{\mathrm{f}},a_{\mathrm{s}},b_{\mathrm{s}}, a_{\mathrm{fs}}, b_{\mathrm{fs}}$, the end-diastolic pressure inside the LV ($\text {EDP}$), and two fibre rotation angles of the RBM for fibre generation: $\alpha _{\mathrm{endo}} \text { and } \alpha _{\mathrm{epi}}$. Each input parameter is considered independently and randomly distributed within the intervals given in Table 3 based on our previous study (Gao et al. 2017a). Two different forms of input parameter distributions are considered in the SA experiments, as will be discussed in Sect. 3.1.

For the I-UQ study, we are interested in inferring the material parameters for fixed fibre angles over a range of different pressures. The reasoning for this choice of experimental design is provided in Sect. 2.4.

**Table 3:** Model Input Parameters based on Gao et al. (2017a)

Input	Unit	Lower	Bounds
			Upper
*a*	kPa	0.1	10
*b*	–	0.1	10
$a_{\mathrm{f}}$	kPa	0.1	10
$b_{\mathrm{f}}$	–	0.1	10
$a_{\mathrm{s}}$	kPa	0.1	10
$b_{\mathrm{s}}$	–	0.1	10
$a_{\mathrm{fs}}$	kPa	0.1	10
$b_{\mathrm{fs}}$	–	0.1	10
$\text {EDP}$	mmHg	4	30
$\alpha _{\mathrm{endo}}$	Degrees	90	0
$\alpha _{\mathrm{epi}}$	Degrees	0	− 90

#### Quantities of interest

We consider four types of end-diastolic response of the LV model as quantities of interest (QoIs) for our experiments: LV cavity volume at end-diastole ($\text {LVV}$), as well as segmental circumferential ($\varepsilon _\text {cc}$), radial ($\varepsilon _\text {rr}$) and longitudinal ($\varepsilon _\text {ll}$) strains since they are widely used in many clinical studies. Definitions for the three strains are

4 $$\begin{aligned} {\left\{ \begin{array}{ll} \varepsilon _\text {cc} = \mathbf{c} \cdot \big ({\mathbf {E}} \, \mathbf{c}\big ), \\ \varepsilon _\text {ll} = \mathbf{l} \cdot \big ({\mathbf {E}}\, \mathbf{l}\big ), \\ \varepsilon _\text {rr} = \mathbf{r} \cdot \big ({\mathbf {E}}\, \mathbf{r}\big ), \end{array}\right. } \end{aligned}$$

where $\mathbf{c}$, $\mathbf{l}$, $\mathbf{r}$ are the unit circumferential, longitudinal and radial directions, and ${\mathbf {E}} = (\mathbf{C}- \mathbf{I})/2$ is the strain tensor with $\mathbf{C}= {\tilde{\mathbf{F}}}^T{\tilde{\mathbf{F}}}$ in which ${\tilde{\mathbf{F}}}$ is the deformation tensor from the end-diastolic phase (the final deformed LV geometry) to the early-diastolic phase (the initial LV geometry). Thus all strains of QoIs are calculated with respect to the end-diastolic phase to be consistent with clinical convention. Note strains of QoIs in (4) are only for post-processing purpose with ${\tilde{\mathbf{F}}} = \mathbf{F}^{-1}$.

To extract the segmental strains in diastole, we first followed the clinical convention by dividing the short-axial images into 6 segments, as visualized in Fig. 3. Normally, 4 short-axial slices are available from the most basal region to the middle of the LV, and dividing each slice into the 6 segments gives a total of 24 segments on the LV. For each of these segments we calculate a spatially averaged strain as defined in (4) to be used in our study for $\varepsilon _\text {cc}$, $\varepsilon _\text {rr}$, $\varepsilon _\text {ll}$, respectively. In the SA study, we choose the segmental circumferential strain, the segmental longitudinal and the radial strains at the inferior lateral segment from the second short-axis slice next to the most basal one, denoted as $\varepsilon ^*_\text {cc}$, $\varepsilon ^*_\text {ll}$, $\varepsilon ^*_\text {rr}$, respectively. Here ‘$^*$’ indicates that the strain is taken from a pre-selected segment. For the I-UQ study (see Sect. 4), all 24 segmental circumferential strains are used.

### Sensitivity analysis

In what follows, we always refer to *sensitivity analysis* (SA) in the sense of *global* sensitivity analysis. For the application of *local* sensitivity analysis to the modelling of the passive LV filling process, see Gao et al. (2015).

We denote the cardiac-mechanic forward model described in Sect. 2.1 as the function *f* that, for a fixed LV geometry, maps a set of input parameter values $X_1, X_2, \ldots X_D$ to associated output quantities of interest, $Y_1, Y_2, \ldots Y_M$:

5 $$\begin{aligned} f(X_1, X_2, \ldots X_D) = (Y_1, Y_2, \ldots Y_M). \end{aligned}$$

In this case, $D=11$, corresponding to the eleven input parameters outlined in Sect. 2.1.4, while the *M* outputs can be chosen to be any of the quantities of interest detailed in Sect. 2.1.5. As detailed in Sect. 3.1, two SA are performed in this paper. The first SA considers $M=4$ output quantities and the second considers $M=2$ outputs. By performing SA on this forward model, we can quantify the influence that the uncertainty in each random input parameter $X_i$ has on the observed variation in any chosen output quantity $Y_j$. The results of the analysis can then be used to identify those inputs that strongly influence output variation, and those inputs that are only weakly influential. Strongly influential parameters can then be prioritized for measurement or estimation from clinical data, while weakly influential parameters can be set to fixed values within their range of uncertainty. We perform SA of the forward model *f* using the approach of Sobol (2001), by computing the *first-order* and *total-effect* Sobol sensitivity indices of the random input parameters. Below, a brief overview of this approach to SA is given. For a comprehensive overview, the reader is directed to Saltelli et al. (2010) and Gramacy (2020, Chapter 8).

The first-order sensitivity index, denoted $S_{ji}$, of input parameter $X_i$ for output $Y_j$ is defined as:

6 $$\begin{aligned} S_{ji} = \frac{{\mathbb {V}}_{X_{i}}\left[ {\mathbb {E}}_{{\mathbf {X}}_{\sim i}}\left[ Y_j \mid X_{i}\right] \right] }{{\mathbb {V}}[Y_j]}, \end{aligned}$$

where ${\mathbb {E}}$ and ${\mathbb {V}}$ are the expectation and variance operators, respectively, and ${\mathbf {X}}_{\sim i }$ is the vector of all random inputs except for $X_i$. The expectation term in the numerator of (6) is called the main-effect function. It returns the expected value of the output $Y_j$ given a value of the $i^{th}$ input $X_i$, after all other inputs have been integrated over. The variance of the main-effect function is then taken, providing a scalar summary of its variation with respect to $X_i$. This result is then standardized with respect to the unconditional output variance, allowing the index to be interpreted as the fraction of the total observed variance in $Y_j$ attributable to varying $X_i$ alone.

The first-order sensitivity index does not account for interaction effects between different input parameters, which can potentially be significant for nonlinear models. For this reason, we also computed the total-effect index, $T_{ji}$, for each input $X_i$, which is defined with respect to output $Y_j$ as:

7 $$\begin{aligned} \begin{aligned} T_{ji}&=\frac{{\mathbb {V}}[Y_j]-{\mathbb {V}}_{{\mathbf {X}}_{\sim i}}\left[ {\mathbb {E}}_{X_i}\left[ Y_j \mid {\mathbf {X}}_{\sim i }\right] \right] }{{\mathbb {V}}[Y_j]}, \\&= \frac{{\mathbb {E}}_{{\mathbf {X}}_{\sim i}}\left[ {\mathbb {V}}_{X_{i}}\left[ Y_j \mid {\mathbf {X}}_{\sim i}\right] \right] }{{\mathbb {V}}[Y_j]}. \\ \end{aligned} \end{aligned}$$

The numerator of the total-effect index gives the difference between the unconditional output variance, and the variance observed in the output once $X_i$ has been accounted for. This is again standardized with respect to the unconditional output variance, meaning that the total-effect index represents the proportion of observed variance in the output that is due to $X_i$, including all possible interaction effects with other input parameters.

The first-order and total-effect indices must be calculated numerically, using evaluations from the forward model. While efficient low-discrepancy Monte-Carlo sampling schemes have been proposed for performing these calculations (Saltelli et al. 2010), the computational expense of each forward evaluation of the cardiac-mechanic model makes this approach impractical in our case. For this reason, we instead followed Melis et al. (2017), Rodriguez-Cantano et al. (2018) and Gramacy (2020, Chapter 8), by calculating the Sobol indices using evaluations from a computationally cheap surrogate model.

### Surrogate modelling

A surrogate model is a statistical model that approximates a computationally expensive forward model, denoted *f*. The surrogate is trained on a dataset ${\mathcal {D}}$ of input-output pairs from the forward model:

8 $$\begin{aligned} {\mathcal {D}} = \left\{ \left( {\mathbf {x}}_i, f({\mathbf {x}}_i) \right) _{i=1}^N \right\} , \end{aligned}$$

where the location of the inputs ${\mathbf {x}}_i \in {\mathbb {R}}^D$ are chosen to densely cover the domain of interest. In the context of the LV model, the expense of each forward simulation means that it is computationally impractical to directly apply the forward model to the calculation of the first-order and total-effect sensitivity indices. However, it is feasible to run batches of training simulations in advance to create datasets of the form of (8), on which surrogate models can be trained. Evaluations from these surrogates can then be used in place of the numerical solver when performing the SA for each of the chosen output quantities of interest, as in Melis et al. (2017).

To construct the surrogate models, we used Gaussian process (GP) regression. GP regression is a Bayesian nonparametric method that is commonly used for the construction of surrogate models (Kennedy and O’Hagan 2001). In Sect. 2.3.1, we give a brief overview of the approach; for a comprehensive description, the reader is directed to the literature (Rasmussen and Williams 2006; Gramacy 2020, Chapter 5)

#### Gaussian process regression

A stochastic process $\{f(x) \mid x \in {\mathcal {X}}\}$ is called a *Gaussian process* if, for any finite input set $x_1, x_2, \ldots, x_n\! \in \! {\mathcal {X}}$, the corresponding outputs ${\mathbf {f}} = (f(x_1), f(x_2), \ldots, f(x_n))^{\varvec{\top }}$ are jointly Gaussian distributed:

9 $$\begin{aligned} p({\mathbf {f}}) = {\mathcal {N}}({\mathbf {m}}, {\mathbf {K}}). \end{aligned}$$

Note that here we use the same notation *f* to refer to the GP random function above, and the forward model (5). The reason for this shared notation will become clear below. A GP is completely defined by its mean function *m* and covariance function, *k*. These are evaluated on the given input set to specify the mean vector ${\mathbf {m}}$ and covariance matrix ${\mathbf {K}}$ of the joint distribution (9) as follows:

10 $$\begin{aligned} {\mathbb {E}}\left( f(x_i)\right)&= m_i = m(x_i), \end{aligned}$$

11 $$\begin{aligned} \text {Cov}\left( f(x_i), f(x_j)\right)&= K_{ij} = k(x_i, x_j). \end{aligned}$$

Note that, in order for ${\mathbf {K}}$ to be a valid covariance matrix, the covariance function *k* must be positive-definite.

To see how GPs can be used to perform regression, consider having observed training data ${\mathcal {D}}$ of the form of (8). The objective of regression is to learn from the training data an estimate of the unknown, true function, so that predictions can be made for the unobserved outputs ${\mathbf {f}}^*$ at a set of *T* test input locations of interest $\left\{ (x^*_j)_{j=1}^T \right\}$. GP regression is performed by assuming that this underlying function is drawn from a GP with specified mean and covariance functions. Then, by definition, the joint distribution $p({\mathbf {f}}^* \!, {\mathbf {f}})$ is a Gaussian of the form of Equation (9). In this framework, the regression task reduces to the problem of finding the conditional distribution $p({\mathbf {f}}^* \! \! \mid \! {\mathbf {f}})$. Using the properties of the multivariate Gaussian, it can be shown that this distribution is also a Gaussian (see Bishop (2006, Chapter 2)):

12 $$\begin{aligned} p({\mathbf {f}}^* \! \! \mid \! {\mathbf {f}}) = {\mathcal {N}}(\varvec{\mu }, \varvec{\varSigma }) \end{aligned}$$

with mean and covariance given by:

13 $$\begin{aligned} \varvec{\mu }&= \mathbf{m }_* + \mathbf{K }_{N*}^{\mathrm {T}} \mathbf{K }_{NN}^{-1}(\mathbf{f } - \mathbf{m }), \end{aligned}$$

14 $$\begin{aligned} \varvec{\varSigma }&= \mathbf{K }_{**}-\mathbf{K }_{N*}^{\mathrm {T}} \mathbf{K }_{NN}^{-1} \mathbf{K }_{N*}, \end{aligned}$$

in which $$\mathbf{K }_{NN}$$ is the $$N \times N$$ covariance matrix found by evaluating the kernel function pairwise on the training data, and $$\mathbf{K }_{**}$$ is defined analogously for the *T* test data points. Similarly, $$\mathbf{m }$$ is the $$N \times 1$$ mean vector for the training data, and $$\mathbf{m }$$ the $$T \times 1$$ mean vector for the test data points. Finally, $$\mathbf{K }_{N*}$$ is an $$N\times T$$ cross-covariance matrix for the training and test data points.

Performing GP regression then requires only the mean and covariance functions respectively to be chosen. In the present study, we used a zero mean function, as all outputs were normalised to mean zero and unit variance before training. Note from the form of (13) that the predictive distribution over any test points of interest will not be equal to zero once we have conditioned on training data. For the covariance function, we used the squared exponential function with separate length scales for each input dimension:

15 $$\begin{aligned} k({\mathbf {x}}_i, {\mathbf {x}}_j) = \sigma _f^2 \; \text {exp}\left( -\frac{1}{2} \sum _{k = 1}^D \frac{(x_{ik} - x_{jk})^2}{\lambda _k^2}\right) + \eta ^2 \delta _{ij} \end{aligned}$$

where $\eta ^2$ is a small nugget term added to ensure numerical stability in the evaluation of (13) and (14). The use of individual length-scales is referred to as automatic relevance determination (ARD), and allows for differing influence of the input dimensions on the output of the GP. The properties of the kernel are governed by its hyperparameters $\sigma _f, \lambda _1, \lambda _2, \ldots \lambda _k, \eta$, and their values can be tuned to allow the GP to best represent the true underlying function. We did this by setting the hyperparameters to those values that maximized the log marginal likelihood of the training data. While fully Bayesian approaches are possible for fitting the hyperparameters, obtaining a point estimate in this manner is the most common approach in the GP literature. This is because the number of hyperparameters is relatively low when compared to flexible parametric models, and for sufficiently large training sets the posterior distributions tend to be highly peaked.

The posterior mean of the GP, which was provided in (13), gives the best estimate for the unknown true function values under quadratic loss. Using this point estimate to compute the Sobol indices allows for efficient calculation that is multiple orders of magnitude quicker than directly using the numerical forward model. Note, however, that in addition to a best point estimate, the GP returns a full posterior probability distribution (12) over the unknown function values. In this paper, we follow the approach detailed in Gramacy (2020, Chapter 8) and, instead of plugging in a single point estimate to compute the Sobol indices, draw an ensemble of samples from the posterior distribution of the GP, and compute the Sobol indices for each sample. The uncertainty in the value of the indices can then be quantified by calculating summary statistics of the resulting ensemble of index values.

### Posterior parameter inference

For a given patient, we assume a fixed, but unknown, set of material parameters. Measurement of these parameters would require mechanical tests on a sample of dissected myocardium, so we will infer these from measurements of circumferential strain and end-diastolic volume obtained in vivo. Parameter estimation for a specific patient requires that we specify suitable physiological conditions—the EDP and fibre angles—which were treated as random variables in the SA study. As discussed in Sect. 2.1.4, we fix the fibre angles for the I-UQ study. This is motivated by their low sensitivity indices for the circumferential strains and LV cavity volume from the SA study, see Sect. 3.4 for details. On the other hand, in vivo estimation of these angles could be available with further development of already existing techniques, i.e. diffusion-tensor MRI (Toussaint et al. 2013; Khalique et al. 2020; Das et al. 2021). This also allows us to focus on the material parameters in a slightly simplified system.

From the model, we obtain 24 circumferential strains, $\mathbf {s}=\{\varepsilon _\text {cc}^{i}, i = 1, \ldots, 24\}$ and a prediction of the end-diastolic volume, $V$. We assume that the measured quantities, ${\mathbf {y}}$, relate to these predictions in the following model:

16 $$\begin{aligned} {\mathbf {y}}=(V,\mathbf {s})+\varvec{\xi }, \end{aligned}$$

where $(V,\mathbf {s})$ is the concatenation of the volume prediction and strain predictions and $\varvec{\xi }$ is a random variable representing the additive noise. Future work could also include segmental radial and longitudinal strains in this model but currently there is no method for obtaining these at high precision from CMR images. For this reason, we choose to leave them out, better resembling what would be feasible with measured data. In previous work (Davies et al. 2019; Noè et al. 2019), this model was assumed to be homoscedastic Gaussian (Gaussian with constant variance), with the standardized volume and strains corrupted by the same level of Gaussian noise. A more accurate representation of reality, however, is to assume that the volume is corrupted by a separate, smaller, variance than the circumferential strains:

17 $$\begin{aligned} y_0=V+\xi _0, \quad \tilde{{\mathbf {y}}}=\mathbf {s}+{\tilde{\varvec{\xi }}}, \end{aligned}$$

where $\xi _0\sim {\mathcal {N}}(0,\sigma _0^2)$ is a random variable representing the additive Gaussian noise on the volume and ${\tilde{\varvec{\xi }}}\sim \text {MVN}({\mathbf {0}},{\tilde{\sigma }}^2 {\mathbf {I}})$ is a random variable representing the additive independent identically distributed (iid) Gaussian noise on the circumferential strains. Given that $\sigma ^2_0$ is the variance of the noise on only one measurement, this parameter is non-identifiable. As a result, we must fix this variance during inference to a value obtained in empirical studies. The assumption that the 24 circumferential strains are all corrupted by Gaussian noise with the same variance ensures that the other variance parameter, ${\tilde{\sigma }}^2$, can be inferred along with the material parameters. The noise model from (17) gives rise to the following log-likelihood function:

18 $$\begin{aligned} \begin{aligned} l({\mathbf {y}}\mid {\varvec{\theta }},{\tilde{\sigma }}^2)=&-\frac{1}{2}\log (\sigma ^2_0)-\frac{1}{2\sigma ^2}(y_0-f_0({\varvec{\theta }}))^2\\&-\frac{24}{2}\log ({\tilde{\sigma }}^2)-\frac{1}{2{\tilde{\sigma }}^2}\sum _{i=1}^{24}(y_i-f_i({\varvec{\theta }}))^2, \end{aligned} \end{aligned}$$

where $f_i({\varvec{\theta }})$ is the *i*th circumferential strain prediction at material parameters ${\varvec{\theta }}$. Note that, in the I-UQ study, ${\varvec{\theta }}$ does not contain all the 8 parameters from the H-O model (1), instead, we will use an SA study to inform a new parametrization of the H-O model where some parameters are held fixed.

We assign a uniform prior on the parameters and, in light of the observed data, update this prior to a posterior distribution for the parameters conditional on the data. For intractable models this update cannot be performed analytically, leading us to use Markov chain Monte Carlo methods for sampling from the posterior distribution of the parameters. Typically, these methods would not be applicable for parameter inference in left ventricle models due to the high computational costs. However, through a statistical surrogate model (see Sect. 2.3) we obtain an approximation to the likelihood in (18) by replacing the expensive evaluation, $f_i({\tilde{{\varvec{\theta }}}})$, with ${\hat{f}}({\tilde{{\varvec{\theta }}}})$ where ${\hat{f}}(\cdot )$ is given by a GP or an alternative regression model that can be trained to efficiently predict the outputs of the simulator. Given that the likelihood is now an approximation of the true function, we incur a bias in our parameter inference that depends on the accuracy of the surrogate model. Methods have been developed to correct these biases using expensive forward simulations (Rasmussen 2003; Conrad et al. 2016) but for our models this would drastically increase the computational costs.

The efficiency of MCMC methods is determined by the proposal mechanism. The simplest and best known approach is the Metropolis-Hastings (MH) algorithm (Hastings 1970). In MH, a move is proposed from *x* to $x'$ based on some proposal distribution, $q(x'|x)$, which is taken to be a normal distribution in the Metropolis algorithm. This move is then accepted or rejected based on an acceptance ratio, satisfying detailed balance. MH works in simple problems, but its efficiency in more complex scenarios can be poor.

More effective proposal mechanisms that take into account the geometry of the posterior surface have been proposed. One example is Hamiltonian Monte Carlo (HMC), which uses gradient information to evolve the system according to Hamilton’s equations using a series of leapfrog steps (see Chapter 5 of Brooks et al. (2011)). More recently the No-U-Turn Sampler (Hoffman and Gelman 2014) was proposed, offering a variant of HMC where the number of leapfrog steps is automatically tuned. This is the method adopted for our uncertainty quantification. These more sophisticated MCMC methods require gradient evaluations and incur greater computational costs for each move in the trajectory, making the use of surrogate modelling essential.

MCMC works by generating a Markov chain with the target distribution as its stationary distribution (Gelman et al. 2013). To assess convergence to this distribution, we use the Gelman-Rubin statistic (Gelman and Rubin 1992) (also known as the potential scale reduction factor (PSRF)). The Gelman-Rubin statistic requires a set of Markov chains initiated from overly dispersed start points (for instance, a Sobol sequence), from which we measure the between and within-chain variances. Using a weighted sum of these variances gives a numerical quantity, for each parameter, that we can use to check for convergence. In this work, 5 chains were initiated from different start points and a PSRF of less than 1.01 was taken to indicate convergence to the stationary distribution.

## SA simulation studies

We have carried out two SA experiments on the H-O model, the results of which are presented in this section. Section 3.1 gives the details of the two experimental studies, before Sects. 3.2 and 3.3 describe how GP surrogate models were trained and validated. Finally, the experimental results obtained using the surrogates are presented and discussed in Sect. 3.4.

### Experimental setup

#### Sensitivity analysis one

In the first numerical experiment, which we refer to as Sensitivity Analysis One (SA1), we perform SA where all eleven input parameters detailed in Sect. 2.1.4 are considered random variables. The analysis is performed for four different output quantities: $\text {LVV}$, $\varepsilon ^*_\text {cc}$, $\varepsilon ^*_\text {ll}$ and $\varepsilon ^*_\text {rr}$. For brevity, each strain value is considered only in the inferior lateral segment at the second short-axial slice from Fig. 3, as the SA results for each strain type are similar across the 24 segmental strain regions. To examine the influence on the SA results of the prior distribution for the eight material parameters, two prior distributions are considered for this experiment. In the first experiment, the material parameters are considered to be independently uniformly distributed between the bounds given in Table 3. We refer to this as the uniform-prior. In the second experiment, the material parameters are considered to be independently uniformly distributed on the *log-scale* between these bounds. We refer to this as the log-uniform prior. For both priors, the non-material parameter inputs: $\text {EDP}$, $\alpha _\text {endo}$ and $\alpha _\text {epi}$, were considered independently uniformly distributed between the bounds stated in Table 3. The computation of the Sobol indices (6) and (7) was performed using the Python package SAlib (Herman and Usher 2017).

#### Sensitivity analysis two

In Sensitivity Analysis Two (SA2), the effect of EDP on the sensitivity scores of the material parameters of the H-O model is explored. Specifically, we fixed the value of EDP to each of the values $\{5, 7.5, 10, \ldots 25\}$ mmHg, and then conducted an emulator-based sensitivity analysis for each fixed pressure value. We carried out this experiment because the results of SA1 considered EDP as a random variable, with a wide range of uncertainty between 4 and 30 mmHg. In practice however, for a given subject, EDP could be a measured value. For this reason, this study examines whether the sensitivity scores of the material parameters vary for different fixed $\text {EDP}$ values. As discussed in Sect. 2.4, currently only $\text {LVV}$ and $\varepsilon _{cc}$ can be measured with high accuracy from cardiac imaging scans (Mangion et al. 2016), so we restrict this analysis to these two output quantities. In addition, because the material parameters are of primary interest in this experiment, we do not consider the two RBM fibre generation parameters as random variables for SA2. Instead, $\alpha _{\mathrm{endo}}$ and $\alpha _{\mathrm{epi}}$ were fixed to the values $60^o$ and $-90^o$ (Lombaert et al. 2011), respectively.

### Surrogate model training

We perform the SA experiments using GP surrogate models, as discussed in Sect. 2.3.1; one surrogate each for the four output quantities under consideration. Constructing these surrogate models requires training data sets of the form of (8) to be created for each output. This in turn requires the *location* and *number* of training inputs to be specified. Given the high computational costs incurred with each forward simulation of the LV diastolic filling process, we seek to choose a set of input points that cover the parameter domain described in Sect. 2.1.4 as efficiently as possible. The simplest approach to selecting the input locations would be to randomly sample points from a uniform distribution over the parameter domain. However, the problem with random sampling is that it can lead to a clustering of points in some regions of the space, leaving other regions unfilled. For this reason, we instead used a Sobol sequence of length 2000 to specify the location of the input points. A Sobol sequence is a quasi-random, low-discrepancy sequence widely used in the design of computer experiments (Fang et al. 2005). As we consider two material parameter priors for the SA, the first 1000 material parameter samples were taken in standard parameter space, and the final 1000 were taken in log-parameter space. We then ran a simulation from each of these input points and extracted the output quantities of interest from each simulation result, yielding a training data set of 2000 input-output pairs on which the GP surrogates for each output QoI could be trained.

**Fig. 4:**
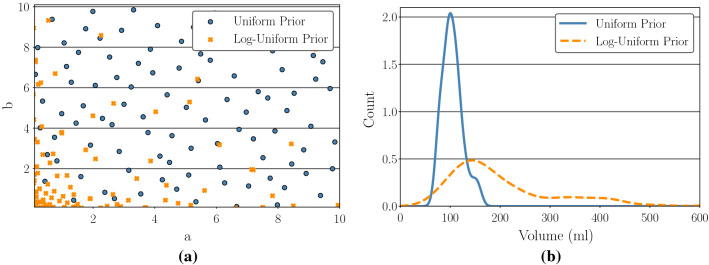
**a** Distribution of first 100 *a*/*b* values for uniform design (blue), and log-uniform design (orange). **b** Density plots for $\text {LVV}$ for simulations run from input points in (**a**)

The effect of the two alternative material parameter sampling procedures is illustrated in Fig. 4a, which plots the first 100 sampled *a* and *b* values in standard space, and the first 100 sampled in log space. By sampling in log space, lower stiffness material parameter configurations are favoured. In this lower stiffness region where the cardiac tissue is softer, the magnitude of the expansion of the left ventricle is more sensitive to small changes in the material stiffness parameters than the case at higher stiffness levels. As a result, the variance observed in the end-diastolic quantities of interest is higher under the log-uniform prior than for the uniform prior. This is illustrated in Fig. 4b, which displays empirical density plots for the simulated $\text {LVV}$ obtained when running from the input points of Fig. 4a. With the log-uniform sampled points, the distribution of $\text {LVV}$ values exhibits a significantly longer tail.

Each GP surrogate was fitted using GPflow (Matthews et al. 2017) in Python with the squared exponential kernel from (15) used to define the covariance structure. As discussed at the end of Sect. 2.3.1, we used optimisation of the marginal likelihood to infer the kernel hyperparameters. When training the GPs, we used the *log* of the eight material input parameters as inputs, rather than the raw values. This approach is similar in principle to the one adopted by Calandra et al. (2016) but with a more context specific transformation. The log transformation accounts for the changing rate of function variation over the input space, making the function more stationary and hence more compatible with the stationary kernel function we use here (see section 4.2.1 of Rasmussen and Williams (2006)).

### Surrogate model validation

By performing the SA experiments with surrogate models rather than the true forward model, we will introduce some error in the calculation of the Sobol indices (6) and (7). It is therefore essential that the accuracy of the surrogates is validated. We did this by evaluating their accuracy on a set of 100 independent test simulations from the forward model. The input locations that the test simulations were run from were found by continuing the Sobol sequence used to generate the training data for a further 100 points. Note that, for the material stiffness parameters, the sequence was run in log space, which favours less stiff material parameter configurations as discussed in Sect. 3.2 above. The accuracy of the surrogates was quantified using the $Q^2$ coefficient. If we denote the true outputs from the simulator as ${\mathbf {f}}^*$, and the predictions of the GP surrogate as ${\hat{\mathbf {f}}}^{\mathbf {*}}$, where ${\hat{\mathbf {f}}}^{\mathbf {*}}$ is set to be the posterior mean in (13), then the $Q^2$-coefficient is defined as:

19 $$\begin{aligned} Q^2 = 1 - \frac{({\mathbf {f}}^* - {\hat{\mathbf {f}}}^{\mathbf {*}})^{\varvec{\top }} ({\mathbf {f}}^* - {\hat{\mathbf {f}}}^{\mathbf {*}}) }{({\mathbf {f}}^* - {\bar{\mathbf {f}}}^{\mathbf {*}})^{\varvec{\top }} ({\mathbf {f}}^* - {\bar{\mathbf {f}}}^{\mathbf {*}}) } \end{aligned}$$

where ${\bar{\mathbf {f}}}^{\mathbf {*}}$ is the vector of the mean value of the test outputs. This definition is analogous to that for the $R^2$ coefficient, except it is calculated on out-of-sample, rather than in-sample data. The $Q^2$-coefficient gives the proportion of the variance in the test outputs that is accounted for by the surrogate model, with values close to one indicating that the surrogate has high predictive accuracy.

### Results

#### Surrogate model verification

The SA experiments were performed using GP surrogate models with squared-exponential covariance functions, as described in Sect. 3.2. For the four output quantities of interest in the SA, $\text {LVV}$, $\varepsilon ^*_\text {cc}$, $\varepsilon ^*_\text {ll}$ and $\varepsilon ^*_\text {rr}$, we validate the accuracy of the surrogates on the 100 test simulations detailed in Sect. 3.3. Table 4 presents the $Q^2$-coefficient for the four outputs, to two decimal places. The $Q^2$ values for $\text {LVV}$, $\varepsilon ^*_\text {cc}$ and $\varepsilon ^*_\text {ll}$ are each 0.98, indicating very strong agreement between the true outputs and the predictions of the surrogate model. The surrogate model for $\varepsilon ^*_\text {rr}$ is less accurate, with a $Q^2$-coefficient of 0.86. Nevertheless, this value still indicates good predictive accuracy, and for this reason, we proceeded to use these trained surrogates to perform the two SA experiments described in 3.1. Additional validation results for the GP emulators are given in Appendix A, including the learned kernel hyperparameter values from Equation (15) and contour plots of the posterior GP mean and standard deviation.

**Table 4:** Surrogate Model Verification Results: $Q^2$-coefficient values for the four output quantities considered for the SA experiments, calculated on a set of 100 independent simulations from the forward model

Output	$Q^2$
$\text {LVV}$	0.98
$\varepsilon ^*_\text {cc}$	0.98
$\varepsilon ^*_\text {ll}$	0.98
$\varepsilon ^*_\text {rr}$	0.86

#### Sensitivity analysis one

**Fig. 5:**
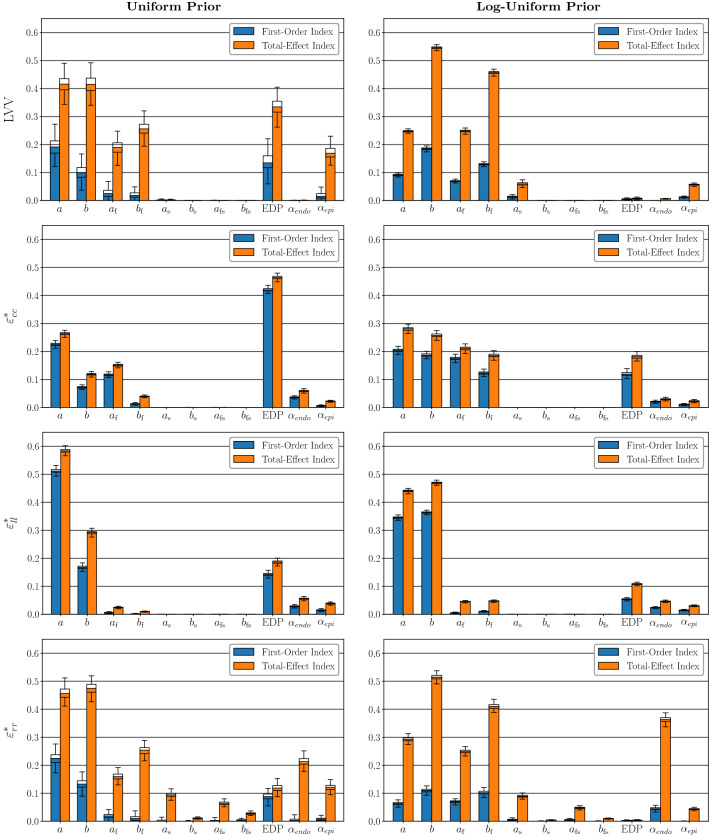
SA1 Results. The left column shows the SA results under the uniform material parameter prior, and the right column shows the results under the log-uniform prior. Each row corresponds to one of the four output QoIs respectively

The results of SA1 are displayed in Fig. 5. All eleven input parameters detailed in Sect. 2.1.4 are assumed to be random variables in this experiment. As discussed in Sect. 3.1, the analyses are repeated for two prior distributions over these inputs; the uniform prior, and the log-uniform prior, where the log-uniform prior favours less stiff material parameter configurations. Four output quantities of interest at end-diastole are considered: $\text {LVV}$, $\varepsilon _\text {cc}^{*}$, $\varepsilon _\text {ll}^{*}$ and $\varepsilon _\text {rr}^{*}$. The four rows of Fig. 5 correspond to each of these output quantities, while the left column shows the results under the uniform prior, and the right column shows the results under the log-uniform prior. The analyses were carried out using the sampling approach detailed in Sect. 2.3. That is, rather than plug in the point estimate (13) from the GP surrogate when numerically evaluating the Sobol indices, 250 samples were drawn from the posterior distribution of the GP (12). The Sobol indices were then evaluated for each sample, resulting in an ensemble of estimated indices. The bars in Fig. 5 correspond to the ensemble mean values, and a boxplot is then overlaid on each bar, showing the uncertainty we have in the index values.

Comparing the two columns of Fig. 5 allows the effect of the two different prior distributions on the SA indices to be assessed. Under the log-uniform prior, more probability mass is placed on less stiff material parameter configurations than is the case for the uniform prior, which in turn leads to a significant tail in the distribution of the magnitudes of the resulting simulated displacements. This effect is illustrated in Fig. 4. Nevertheless, the SA results under the two priors indicate qualitatively reasonable agreement. That is, inputs that have close to zero influence under one prior tend to have close to zero influence under the other, with the same agreement observed for highly influential inputs. The exception to this is the influence of $\text {EDP}$, which exhibits significantly less influence on each QoI under the log-uniform prior. This issue is discussed in more detail below.

Considering the sensitivity indices observed for the material stiffness input parameters, we can see that *a*, *b*, $a_{\mathrm{f}}$ and $b_{\mathrm{f}}$ are clearly the most influential on the output quantities of interest. For all analyses except $\varepsilon ^*_{cc}$ under the uniform prior, either *a* or *b* has the highest total-effect index, with the effect of *a* tending to be larger under the uniform prior, while *b* is generally more influential under the log-uniform prior. Parameters $a_{\mathrm{f}}$ and $b_{\mathrm{f}}$ tend to have lower, but still significant sensitivity indices, and for $\text {LVV}$ under the log-uniform prior they are the third and second most influential inputs, respectively. By contrast, the total-effect sensitivity indices are always lower for $a_{\mathrm{s}}$, $b_{\mathrm{s}}$, $a_{\mathrm{fs}}$ and $b_{\mathrm{fs}}$ than the other material parameters, for each combination of output quantity and material prior distribution, and in many cases their indices values are very close to zero. Given that these four parameters have very little influence on the variation in the model outputs, if we now consider the inverse problem of trying to estimate their values given from noisy experimental data, they will be essentially non-identifiable. For this reason, in subsequent experiments we fix them to values from the literature (Gao et al. 2017b). $\text {EDP}$ has significant first-order and total-effect sensitivity indices for each output quantity under the uniform prior. However, under the log-uniform prior, the influence of $\text {EDP}$ is lower for all outputs. For $\text {LVV}$ and $\varepsilon ^*_\text {rr}$ in particular, the influence of $\text {EDP}$ is close to zero. This is because the log-uniform prior places more weight on low material stiffness parameter configurations, as illustrated in Fig. 4a, aggregated in the left bottom corner. For sufficiently low material stiffness parameter values, the magnitude of $\text {LVV}$ and $\varepsilon ^*_\text {rr}$ values at the end of diastole are very high, across the entire range of $\text {EDP}$ values considered in this study. As a result, the influence of $\text {EDP}$ on observed variance for $\text {LVV}$ and $\varepsilon ^*_\text {rr}$ is suppressed by the large influence of the material stiffness parameters, resulting in Sobol indices that are close to zero. By contrast, $\varepsilon ^*_\text {cc}$ and $\varepsilon ^*_\text {ll}$ do not exhibit the same level of skewness under the log-uniform prior, meaning that the influence of $\text {EDP}$ on these two QoIs remains significant. For both choices of prior, the fibre orientation angles $\alpha _{\mathrm{endo}} \text { and } \alpha _{\mathrm{epi}}$ tended to have a low, but still clearly non-zero, impact on the end-diastolic output quantities. The exception to this was $\varepsilon ^*_\text {rr}$ under the log-uniform prior. Here, $\alpha _\text {endo}$ shows a small first-order sensitivity index, but a large total-effect index.

Note that, in response to a reviewer’s suggestion, we have also included results of SA1 for an additional QoI, the apical torsion, in Appendix B.

#### Sensitivity analysis two

In SA2, $\text {EDP}$ was not assumed to be a random variable. Instead, a range of fixed $\text {EDP}$ values (5, 7.5, … 25) mmHg were considered, and a separate SA was carried out for each fixed value. The objective of this experiment was to quantify how this fixed $\text {EDP}$ value impacts the Sobol indices of the material parameters. This is because, for a given subject, $\text {EDP}$ will not be a random variable over the range [4, 30] mmHg as in SA1, but will be a fixed value, which can in principle be measured. Two output QoIs are analysed; $\text {LVV}$ and $\varepsilon ^*_\text {cc}$. Since the material parameters $a_{\mathrm{s}}$, $b_{\mathrm{s}}$, $a_{\mathrm{fs}}$ and $b_{\mathrm{fs}}$ exhibited very low influence on $\text {LVV}$ and $\varepsilon ^*_\text {cc}$ in SA1 (see Fig. 5), we fix these parameters in this study and concentrate our analysis on the material parameters *a*, *b*, $a_{\mathrm{f}}$ and $b_{\mathrm{f}}$. The fixed values are those published in (Gao et al. 2017b), namely $a_{\mathrm{s}}=0.69$ kPa, $b_{\mathrm{s}}=1.11$, $a_{\mathrm{fs}}=0.31$ kPa and $b_{\mathrm{fs}}=2.58$. In addition, because the material parameters are of primary interest here, the RBM parameters $\alpha _{\mathrm{endo}}$ and $\alpha _{\mathrm{epi}}$ are also considered fixed, to the values $60^o$ and $-90^o$, respectively.

**Fig. 6:**
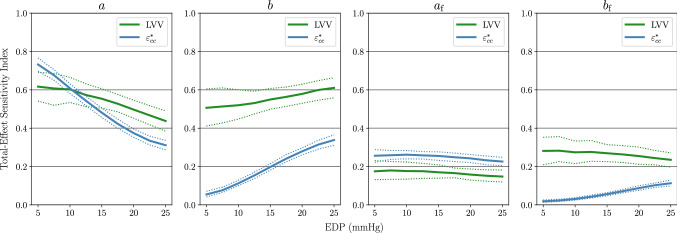
SA2 Results under the uniform material parameter prior. The dashed lines indicate 95% credible intervals

**Fig. 7:**
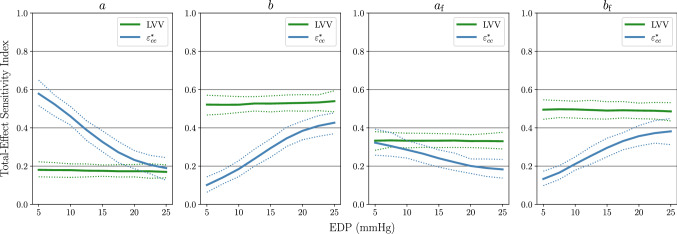
SA2 Results under the log-uniform material parameter prior. The dashed lines indicate 95% credible intervals

The results of this experiment are displayed in Fig. 6 for the uniform prior and Fig. 7 for the log-uniform prior. As in SA1, the GP sampling approach discussed at the end of Sect. 2.3 was used to generate an ensemble of total-effect Sobol index values at each fixed $\text {EDP}$ value. The solid lines in Figs. 6 and 7 show the ensemble mean values, while the dashed lines indicate 95% credible intervals (CIs). These results exhibit stability under the different choices of prior. That is, the effect of $\text {EDP}$ on the Sobol indices of each material parameter is qualitatively similar for both the uniform and log-uniform prior distributions. For $\text {LVV}$ under the uniform prior the effect of *a* decreases with rising $\text {EDP}$, while the effect of *b* increases slightly, whereas the sensitivity scores of $a_{\mathrm{f}}$ and $b_{\mathrm{f}}$ do not exhibit large variation as a function of $\text {EDP}$. Under the log-uniform prior, the influence of all four material parameters remain constant as a function of $\text {EDP}$. This is consistent with the results of SA1, where the Sobol indices of $\text {EDP}$ for $\text {LVV}$ were found to be close to zero with the log-uniform prior. For $\varepsilon ^*_\text {cc}$, the effect of $\text {EDP}$ on the sensitivity indices of the material parameters tends to be larger than for $\text {LVV}$. Specifically, the total-effect scores of *b* and $b_{\mathrm{f}}$ on $\varepsilon ^*_\text {cc}$ *increase* as $\text {EDP}$ rises, whereas the total-effect scores of *a* and $a_{\mathrm{f}}$ *decrease* with rising $\text {EDP}$.

## I-UQ simulation studies

We now use an I-UQ study to build on the results of the SA. The details of the study to be carried out are given in Sect. 4.1, the implementation and results of which are discussed in Sect. 4.2 and Sect. 4.4, respectively.

### Experimental setup

#### Inputs and outputs of the emulator

As explained in Sect. 2.4, only the circumferential strains and end-diastolic volume were included in the outputs of the I-UQ emulator. Motivated by the results of Sect. 3.4.2, we adopted a reduced parameterization of the H-O model (1) where $a_{\mathrm{s}}, \,b_{\mathrm{s}}, \,a_{\mathrm{fs}}\text { and } b_{\mathrm{fs}}$ were held fixed to values obtained from the literature (Gao et al. 2017b). Additionally, the fibre angles were held fixed at $\alpha _\text {endo}=60^o$ and $\alpha _\text {epi}=-90^o$, as discussed in Sect. 2.4. The resulting five dimensional input space, containing $a,b,a_{\mathrm{f}},b_{\mathrm{f}}$ and EDP, is identical to the one considered in Sect. 3.4.3.

#### Test data generation

For the I-UQ, we require a set of test data to be used as observations for the posterior inference. These were generated by running the simulator from Sect. 2.1 at various different material parameter configurations. This is identical to the procedure for generating the training data of the emulator, with some discrepancy introduced in the form of additive Gaussian noise as outlined in Sect. 2.4. A total of 100 test simulations were obtained at EDPs of 5,10,15,20 and 25 mmHg. For each EDP, the same 100 unique material parameter configurations were used for the simulations, generated from a log-Uniform distribution with bounds 0.1 and 10. Out of the 100 simulations one led to an error in the simulator as a result of excessive distortion of the LV mesh, causing numerical instabilities and unconverged solutions. For this reason, the final test set contained 99 data points. Of interest is I-UQ in the presence of noisy data, so Gaussian noise with standard deviation 5 ml was added to the simulated volumes and Gaussian noise with standard deviation 0.03 was added to the circumferential strains, motivated by results in the literature (Zhang et al. 2021). The volume standard deviation was informed by an empirical study where different operators independently extracted the LV geometry from the CMR scan using the same reconstruction approach. The use of different noise variances on volumes and circumferential strains is consistent with the noise model in (17).

### Surrogate model training

As discussed in Sect. 2.4, the computational costs of the simulator make statistical emulation (see Sect. 2.3) a necessity for I-UQ in any reasonable time frame. For each of the 25 outputs (24 circumferential strains and end diastolic volume) we build an independent GP emulator, which requires a set of training data. The design for statistical emulation was discussed in Sect. 3.2 and here we use a Sobol sequence in log material parameter space. In total, 2000 training simulations were used and the GP emulator was trained over the joint space of pressure and log material parameters.

### Surrogate model validation

The surrogate model was validated on a set of 100 points generated from a Sobol sequence in log material parameter space. The $Q^2$ metric, which was discussed in Sect. 3.3, was used with the results provided in Table 5. Due to the reduction in dimension of the emulator input space the performance is slightly better than with the SA emulator in Table 4.

**Table 5:** Surrogate Model Verification Results: $Q^2$ coefficient value for the two outputs considered for the I-UQ experiments, calculated on a test-set of 100 simulations from the forward model. The values are rounded to three digits

Output	$Q^2$
$\text {LVV}$	1.00
$\varepsilon _\text {cc}$	1.00

### Results

#### I-UQ results in parameter space

**Fig. 8:**
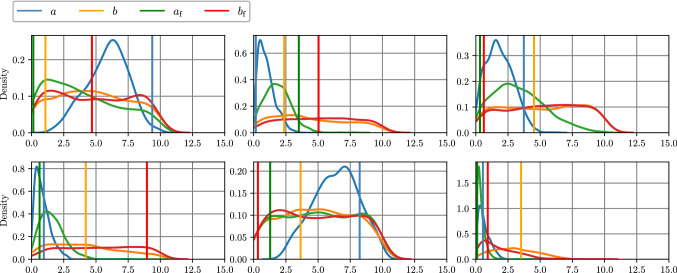
Example posterior density plots obtained from the MCMC samples of the material parameters. Each subplot provides the marginal posterior densities for a separate test case, with the vertical lines showing the ground truth parameter value

**Fig. 9:**
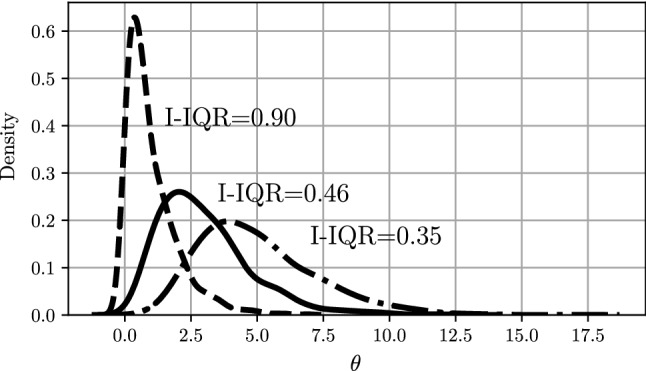
Demonstrating the I-IQR metric. For more peaked distributions (lower uncertainty), the I-IQR is larger and indicates improved practical identifiability. These distributions are purely for demonstrative purposes

**Fig. 10:**
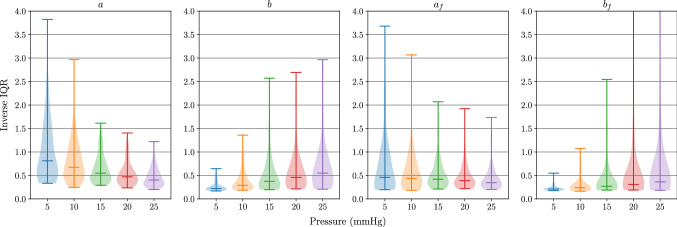
Distributions of I-IQRs of marginal posterior distributions, conditional on data obtained at each of the five different EDPs

**Fig. 11:**
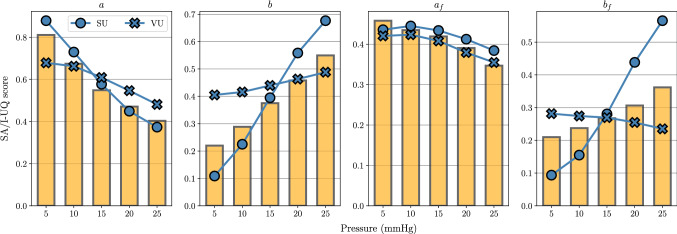
A comparison of SA2 and I-UQ. The I-UQ results are presented by orange bars showing the median I-IQR from the 99 test cases at each pressure. The SA2 results under the uniform prior (strains=SU and volume=VU) are provided as trend plots, allowing us to see the agreement between the results of the two studies

Of interest is the certainty with which we can infer the parameters and how this is affected by EDP. For each of the 99 test cases at all five values of pressure, we sampled the material parameters conditional on the synthetic observed data, generated according to Sect. 4.1.2. The MCMC samples were obtained in the joint space of the four material parameters, but to summarize the results we will consider the marginal posterior of each of the four parameters. Several example marginal posterior density plots are found in Fig. 8. These plots highlight the difference in the practical identifiability of the material parameters. In particular, the distributions for *a* and $a_{\mathrm{f}}$ tend to be more peaked than those of *b* and $b_{\mathrm{f}}$. In the case of high practical identifiability, we expect a more peaked distribution, which can be quantified using the interquartile range (IQR). Taking the inverse of the IQR (denoted as I-IQR) provides a quantity that is analogous to the sensitivity index used in the SA, with large values indicating better practical identifiability of the parameters, as demonstrated in Fig. 9. Since we have 99 different test cases, a set of 99 different I-IQR values at each of the five different pressure values is obtained, giving us five different distributions of I-IQR values as plotted in each subplot of Fig. 10. These are plotted in the form of violin plots, which are similar to boxplots but provide extra information in the form of a kernel density estimator (indicated by the shape of the violin). In the plots of *a* and $a_{\mathrm{f}}$, we see the general trend of the I-IQR decreasing as EDP increases. For *b* and $b_{\mathrm{f}}$, the I-IQR of the marginal posterior distribution increases as EDP increases, suggesting the practical identifiability improves. The change in I-IQR is greater for *a* and $b$ than $a_{\text{f}}$ and $b_{\mathrm{f}}$. These results are in general agreement with the results of SA2 (see Sect. 3.4.3), as shown in Fig. 11.

**Fig. 12:**
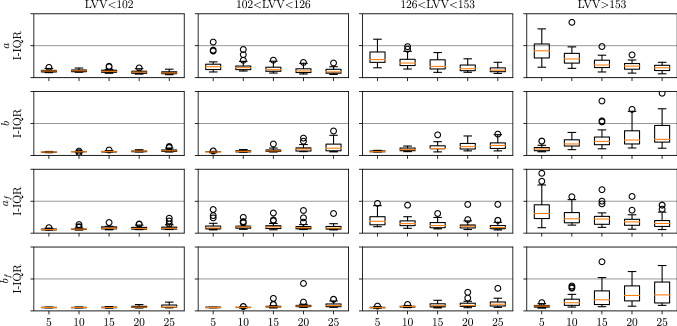
We can split up the I-IQR distributions based on LVV and consider the changes in the distribution of I-IQR with pressure and LVV. Each subplot shows the I-IQR of the marginal posterior for a particular material parameter (per row) for parameter configurations that give a simulated LVV at pressure 10 mmHg in a particular range (per column). For instance, the subplot in the second column from the left of the second row from the top provides the distribution of I-IQR values of the marginal posterior distributions of *b* for test parameter configurations with simulated LVV between 102 and 126 ml at pressure 10 mmHg. The limits of the vertical axes are all the same and have been removed because they are not required for interpretation of the plots

In Fig. 10, we saw the change in the spread of $p(I_\theta |P)$ over *P*, where $I_\theta$ is the I-IQR of the marginal posterior distribution of $\theta$ and *P* is EDP. For *a* and $a_{\mathrm{f}}$, the spread of this distribution decreases while for *b* and $b_{\mathrm{f}}$, the spread increases as we increase *P*. In Fig. 12, we take this a step further and consider the distribution $p(I_\theta |P,{\mathcal {V}})$ where ${\mathcal {V}}$ is the LVV, simulated at pressure 10 mmHg. To do so, we arrange the test cases in bins according to their LVV, with:

$$\begin{aligned}&\bullet v_1: \text {LVV}<102 \,\,&\bullet v_2 : 102<\text {LVV}<126\\&\bullet v_3: 126<\text {LVV}<153 \,\,&\bullet v_4 : \text {LVV}>153.\\ \end{aligned}$$

These values were selected using the 0.25, 0.5 and 0.75 quantiles of the test set LVVs (at EDP=10 mmHg). The *i*th column of plots show the distributions $p(I_\theta |P,{\mathcal {V}}=v_i)$ over the range of pressures. That is to say that within subplot *i*, the different boxes show the distribution $p(I_\theta |P,{\mathcal {V}}=v_i)$, with the EDP indicated by the labels of the horizontal axes in the bottom row. Therefore, if we wish to compare the distributions $p(I_a|P=15,{\mathcal {V}})$ over varying ${\mathcal {V}}$, we should compare the 3rd box in each subplot of the first row of Fig. 12.

All the distributions in the first column of boxplots (corresponding to LVV<102 ml) have a low median value and very little spread. This indicates the difficulty of inferring the parameters when the material is very stiff. As we increase ${\mathcal {V}}$, the I-IQR gradually moves away from zero and the pattern in $p(I_\theta |P,{\mathcal {V}}=v_i)$ begins to resemble the pattern we saw in Fig. 10. These plots suggest that the value of the parameters, which influences the LVV, impacts their identifiability. This feature will now be explored in more detail.

**Fig. 13:**
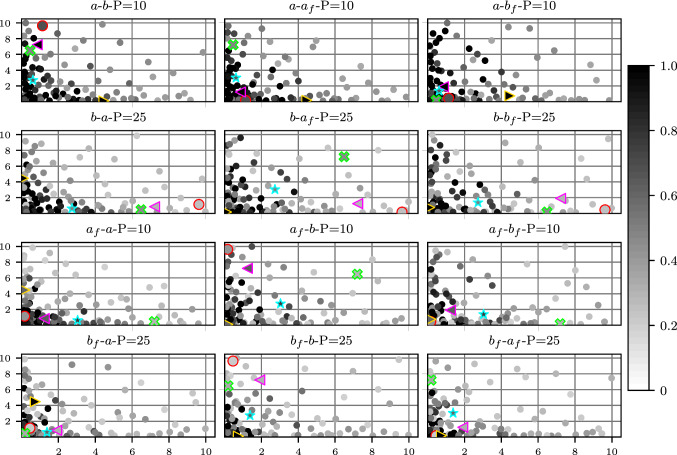
Scatter plot of test points in 2D, coloured based on the I-IQR of the marginal posterior distribution. The title of each plot, $x-y-\text {P}=z$ gives the parameter of the horizontal axis (x), the parameter of the vertical axis (y) and the end-diastolic pressure (P) at which the parameters are inferred (z). The points are shaded based on the I-IQR of the marginal posterior distribution of parameter *x*. Several points are highlighted (by colour and shape) to be looked at more extensively in proceeding visualizations in Fig. 14, corresponding to the five configurations listed in Table 6. All subplots share the same bounds on the vertical and horizontal axes so these are only provided for the outer plots. Units: $\text {P}$ (mmHg), *a* (kPa), $a_\text {f}$ (kPa), *b* (unitless), $b_\text {f}$ (unitless)

Figure 13 shows 2D projections of the 99 test inputs from Sect. 4.1.2. These 2D projections show the value of a primary parameter (the first parameter in the plot title) in the horizontal axis and a secondary parameter (the second parameter in the title) in the vertical axis. The shading of the points indicates the value of the I-IQR of the marginal posterior density of the primary parameter, where the parameters have been inferred conditional on the test data obtained by simulating to EDP as indicated by the title of the subplot. In general, if the ground truth value of a parameter is large (moving left to right on the horizontal axis of a subplot) the I-IQR of the marginal posterior distribution is small. Inconsistency in the pattern of I-IQR values suggests there are other factors influencing the I-IQR values and this will now be explored with several specific test cases.

**Fig. 14:**
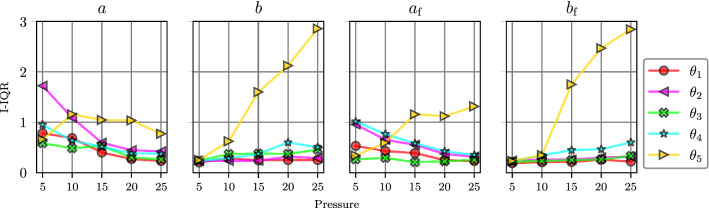
Plotting the change in I-IQR of the marginal posterior distributions over different EDP (mmHg) range for the test cases highlighted in Fig. 13. The colours and shapes of the symbols match those in Fig. 13, and the *x*-axis represents various EDP range

To display some of the effects of parameter values on the I-UQ, several examples are highlighted in Fig. 13. The different edge colours and shapes allow us to see the value of the material parameters for 5 different configurations, which are also given in Table 6. The results for these configurations are considered in more detail in Fig. 14, where the colour and shape of the scatter points match those in Fig. 13. ${\varvec{\theta }}_4$ gives an example where all parameters clearly follow the general pattern seen in Fig. 10. In the case of ${\varvec{\theta }}_1$ and ${\varvec{\theta }}_2$, the large value of *b* contributes to a low I-IQR of $b_{\mathrm{f}}$ at all pressures. This effect of *b* on the inference of $b_{\mathrm{f}}$ at pressure 25 mmHg is also seen in the $b_{\mathrm{f}}-b-$P=25 plot in Fig. 13, where all points in the upper left corner ($b_{\mathrm{f}}$ small and *b* large) are lightly coloured. In ${\varvec{\theta }}_3$, we see an example where despite *a* being small, the I-IQR is fairly small. This shows the influence *b* and $a_{\mathrm{f}}$ can have on the identifiability of *a*. When we push the model to extreme configurations, such as ${\varvec{\theta }}_5$ where *a* is large and all other parameters are small, we get strange patterns in the I-IQR distributions. In this case, the I-IQR of *b* and $b_{\mathrm{f}}$ is huge at high pressure and the I-IQR of $a_{\mathrm{f}}$ increases as pressure increases. This last feature is only observed in these more extreme cases, while a marginal posterior distribution for *b* and $b_{\mathrm{f}}$ with large I-IQR relies on these parameters being very small. Overall, these different examples show the dependence of the parameter identifiability on the values of the other parameters. In particular, this leads to a large spread of the distribution of I-IQRs of *a* and $a_{\mathrm{f}}$ at low pressure and *b* and $b_{\mathrm{f}}$ at large pressures. A further analysis of the I-IQR changes over parameter space is provided in Appendix C, see Fig. 19 for details.

**Table 6:** The four parameter configurations being considered in greater detail

Config	*a* (kPa)	*b*	$a_{\mathrm{f}}$ (kPa)	$b_{\mathrm{f}}$
${\varvec{\theta }}_1$	1.13	9.63	0.16	0.45
${\varvec{\theta }}_2$	0.85	7.22	1.24	1.91
${\varvec{\theta }}_3$	0.46	6.48	7.20	0.18
${\varvec{\theta }}_4$	0.62	2.73	3.04	1.38
${\varvec{\theta }}_5$	4.46	0.10	0.18	0.75

#### I-UQ results in stress–stretch space

Any interpretation of inference results in terms of summaries of marginal posterior distributions is limited by the correlations that exist between the material parameters. In an attempt to overcome this, we also consider the inferred tissue properties in stress–stretch space, where we simulate the uni-axial stretch of a myocardial strip under a given set of material parameters of the H-O model (1). This is done by virtually stretching a myocardial strip along the myocyte and sheet directions in a uni-axial manner. In detail, two myocardial strips are considered here, one is stretched along the myofibre direction and the other one is stretched along the sheet direction with the stretch $\lambda = {l}/{L}$, in which *l* and *L* are the current and the reference lengths of the same myocardial strip. Finally the corresponding stress can be calculated using the H-O model by assuming homogeneous deformation occurring in the entire strip. The interested reader can refer to (Guan et al. 2019) for more details. From a posterior sample of material parameters obtained using MCMC, we can determine a posterior distribution of stress–stretch curves, which is repeated for each of the 99 test cases at each of the five pressures. These distribution of curves are obtained by simulating the deformation of a sample of tissue as characterized by the H-O model (1). Repeating for each sample from the posterior distribution of parameters gives a posterior distribution of stress–stretch curves.

**Fig. 15:**
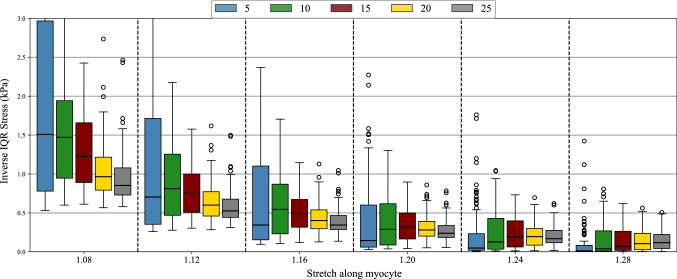
Representing our uncertainty about the tissue properties at different EDPs (in mmHg). From each posterior distribution of material parameters, we obtain a distribution of stress-stretch curves (one curve for each sample). At different stretch locations, we calculate the inverse interquartile range of the stress distribution. Based on the 99 test cases, we get a distribution of these inverse interquartile ranges as found in the boxplots. This can be repeated for test data at different end diastolic pressures to assess the certainty of our estimation of the tissue properties at different pressures

**Fig. 16:**
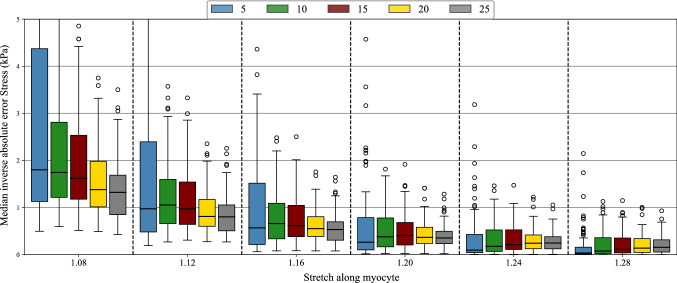
The error in the inferred tissue properties at different EDPs (in mmHg). From each posterior distribution of the material parameters, we obtain a distribution of stress–stretch curves (one curve for each sample). We possess the ground truth stress-stretch curves and can obtain the median inverse absolute error in the distribution of stress–stretch curves. Based on the 99 test cases, we get a distribution of these median inverse absolute errors. This can be repeated for test data at different end diastolic pressures to assess the accuracy of our estimation of the tissue properties at different pressures

**Fig. 19:**
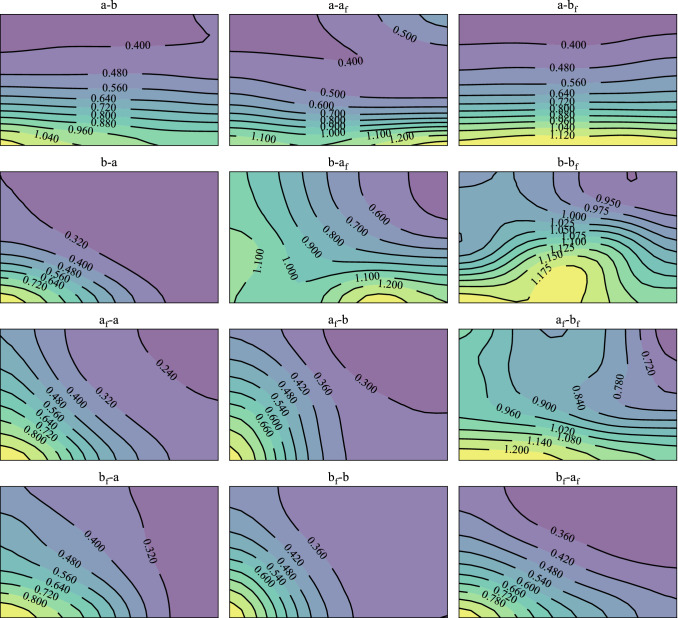
How the I-IQR in posterior samples changes as we vary the position in input space. For each 2-dimensional subspace, the other two parameters are fixed equal to 1 during synthetic data generation

Firstly, we are interested in the uncertainty in the inferred tissue properties, which will be measured using the I-IQR of the stress distribution at given levels of stretch. From the 99 test cases, we get a distribution of I-IQR at each level of stretch, as shown in Fig. 15. The value decreases for all end diastolic pressures as the stretch location increases but the rate of this decrease is different for the different end diastolic pressures. This means that at low stretch the lowest uncertainty in the stress distribution was achieved using low pressure data (higher I-IQR scores, in general) while at high stretch, the lower uncertainty was achieved using high pressure data. The median inverse absolute error, plotted in Fig. 16, shows a similar pattern: at low stretch, the median inverse absolute error stress is highest for pressure 5 and lowest for pressure 25. As we increase the stretch, the pattern gradually reverses.

## Discussion

The objective of this paper has been to perform SA and I-UQ experimental studies of the passive mechanics of the left ventricle using the H-O myocardial model. As illustrated in Fig. 2, these analyses are complementary; the SA quantifies how the structure of the model affects observed data (forward modelling effect), while the I-UQ quantifies how well-observed data can be used to identify the model structure (inverse modelling effect). A total of eleven model input variables were considered across all studies: the eight material parameters of the H-O model, $\text {EDP}$, and two inputs to the RBM fibre generation algorithm. In addition, four different output quantities of interest were considered in total: $\text {LVV}$, $\varepsilon ^*_\text {cc}$, $\varepsilon ^*_\text {ll}$ and $\varepsilon ^*_\text {rr}$, all measured at end-diastole. These input and output quantities are discussed in Sects. 2.1.4 and 2.1.5 , respectively. All experiments were performed with respect to the left ventricular geometry of a healthy volunteer. The SA experiments focused on assessing how uncertainty in each of the *a-priori* uncertain input variables accounted for the uncertainty observed in the output quantities of interest. The I-UQ analysis then built on the results of the SA, to quantify how identifiable the most important material stiffness parameters were from noisy data, with emphasis on quantifying how these identifiability levels were affected by changing $\text {EDP}$ values.

LV diastolic dysfunction, characterized by impaired LV relaxation and increased myocardial stiffness, is one of the main underlying reasons of heart failure with preserved ejection fraction syndrome (Zile et al. 2004), consisting half of the heart failure cases (Owan et al. 2006). LV diastolic dysfunction is also very common after myocardial infarction with reduced ejection fraction (Søholm et al. 2016). Recent research in this area includes (Zhang et al. 2021; Peirlinck et al. 2019). Thus reliable and non-invasive inference of diastolic function using routinely available clinical data is highly needed. Our study is the first to perform a comprehensive global sensitivity analysis and I-UQ of the H-O model in LV passive biomechanics. The inference framework detailed in this study is not limited to passive LV mechanics, but can be applied in other contexts such as active contraction and electrophysiology, for example. The clear takeaway from the results of the SA is that the material parameters *a*, *b*, $a_{\mathrm{f}}$ and $b_{\mathrm{f}}$ are of primary importance to the four output quantities of interest considered, of which *a* and $a_{\mathrm{f}}$ can be fairly inferred using in vivo data. While the relative importance of these parameters varies under the two different prior distributions, Fig. 5 shows that they are consistently the most important inputs for each QoI. In contrast, the material parameters $a_{\mathrm{s}}$, $b_{\mathrm{s}}$, $a_{\mathrm{fs}}$, $b_{\mathrm{fs}}$ have either zero or close to zero influence for each output quantity and prior distribution configuration.

We see the small influence as justification for a low-order re-parameterization of the H-O model, where these parameters are set to fixed values, rather than discarding the two related terms in Equation (1), since such reduction may not fit the experimentally measured stretch-stress data well (Holzapfel and Ogden 2009; Guan et al. 2019). Future studies can focus on the clinical significance of those 4 parameters (*a*, *b*, $a_{\mathrm{f}}$, $b_{\mathrm{f}}$), and develop new imaging techniques to improve parameter identifiability, such as MR elastography (Troelstra et al. 2021).

By using the Guccione myocardial model in LV diastolic filling, Rodriguez-Cantano et al. (2018) showed that the general stiffness parameter *C* has the highest sensitivity for their chosen quantities of interest, around 70%, much higher than other constitutive parameters. They further found that the total Sobol indices for the parameters associating with the fibre direction, the sheet direction and the shear response were clearly not zero. Similar results were reported in their subsequent study (Campos et al. 2019). While we found that the parameters associating with the sheet direction and shear response, in general, have very low sensitivity, but the parameters for the isotropic response and fibre reinforcement have high sensitivity, see Fig. 5. Those different findings may be due to different constitutive laws, QoIs and parameter ranges used in the two studies.

Computational studies have shown that myofibre architecture, such as fibre angles, can have a substantial impact on LV mechanics, but not the sheet angle (Wang et al. 2013; Palit et al. 2018; Guan et al. 2021). Thus in this study, we only included the fibre rotation angles in the inputs. These angles were found to generally have a low influence on the observed variations in the output quantities, but not zero in general, especially for radial strains. Similar results have been reported in Rodriguez-Cantano et al. (2018). Fibre angles have been found to potentially have an even higher impact on myocardial active contraction (Campos et al. 2019). In the literature, non-rotationally symmetric distributions (Holzapfel et al. 2019), such as $\pi$-periodic Von Mises distributions, have been used to model fibre dispersion in the myocardium (Guan et al. 2021). Due to limited measured data for describing local fibre distribution variations, we also have not included the uncertainty in fibre dispersion. Future sensitivity studies shall incorporate both uncertainties in the fibre rotation angles (i.e. a truncated Karhunen-Loeve expansion (Rodriguez-Cantano et al. 2018)) and fibre dispersion, in particular for myocardial contraction.

LV cavity pressure usually needs to be measured in an invasive way, thus it is not available for most cardiac patients. Instead, many existing studies rely on population-based $\text {EDP}$ (Gao et al. 2017a; Genet et al. 2014). LV $\text {EDP}$ was found to have high importance for the uniform prior, while its influence declined under the log-uniform prior, as discussed in Sect. 3.4. The high sensitivity of $\text {EDP}$ during the diastolic filling highlights the need for accurate measurement of $\text {EDP}$, in particular by using non-invasive approaches. One such recent development is using 4D cardiac CMR flow imaging to estimate realistic ventricular relative pressure (Marlevi et al. 2021). We also see from the results of SA2 in Figs. 6 and 7 respectively that the influence of the material parameters *a*, *b*, $a_{\mathrm{f}}$ and $b_{\mathrm{f}}$ on circumferential strains can vary significantly as a function of the value of $\text {EDP}$ when it is considered a known input. This has important implications when we consider the inverse problem of trying to estimate these parameters from experimental data for a given patient with a measured LV $\text {EDP}$.

Considering the results of the I-UQ study, in Fig. 10 we found that, as we vary $\text {EDP}$, the uncertainty in the marginal posterior distributions changes as follows: the uncertainty of *a* and $a_{\mathrm{f}}$ *increases* as we increase pressure while the uncertainty of *b* and $b_{\mathrm{f}}$ *decreases*. This uncertainty was measured using the I-IQR metric (see Fig. 9). This trend in identifiabilities over $\text {EDP}$ agrees with the SA results from Fig. 6, especially if we consider the fact that our inverse estimation is based on a set of measurements containing 24 circumferential strains and only one end-diastolic volume ($\text {LVV}$), meaning that the circumferential strains carry extra weight in the likelihood function from (18). These results are also in line with our physical interpretation of the parameters, which is that *a* and $a_{\mathrm{f}}$ govern low stretch behaviour of the myocardium while *b* and $b_{\mathrm{f}}$ govern high stretch behaviour in the nonlinear regime.

We found that there is quite a large change in the spread of the distribution of I-IQR values, suggesting variation in the identifiability of the parameters. In particular, parameter inference in the case of large material parameters tends to lead to lower I-IQR values. This is a result of the non-stationarity discussed in Sect. 3.2, leading to improved parameter identifiability in the region where the function varies more rapidly. For emulation purposes, a log-transformation was proposed to account for this effect in the GP model.

The interpretation of inference results in terms of marginal posterior distributions is confounded by the strong coupling that exists between the parameters of the H-O model. To overcome this, we also considered our results in stress-stretch space, providing a more direct measurement of tissue behaviour that takes into account the entire set of material parameters. Figure 15 showed the change in distributions of inverse IQRs for different $\text {EDP}$ at different uni-axial stretches. As we expect, the uncertainty in stress locations increases as the stretch increases and the pressure that provides the lowest uncertainty in the stress distribution increases. This pattern in the pressures is intuitive, since larger pressures give us information about the behaviour of the tissue at higher stretch locations. We also used the stress–stretch curves to assess the accuracy of our inference in Fig. 16. These results show the same behaviour as the IQR results, with high pressure data giving lower error in the high stretch region.

## Future work

In this Section, we describe a number of ways in which future work could expand on the analyses presented in this manuscript.

For our experiments, we have used Gaussian process surrogate models to directly emulate the chosen output QoIs. An alternative emulation approach would have been to construct a surrogate model for the displacement of the LV in diastole. Any output QoI, such as LVV, could then be calculated from the deformed geometry predicted by the emulator. The primary advantage of the approach we have taken in this work is prediction speed. By directly emulating the real-valued output QoIs, we significantly reduce the number of operations required to make a prediction compared to a displacement-based emulation approach. This in turn allows the SA and I-UQ experiments to be performed significantly faster. In addition, we show in Sections 3.4.1 and 4.3 that the direct GP emulators display strong accuracy with respect to the forward model. We believe, however, that emulation of the LV displacement field using Graph Neural Networks (GNNs) (Murphy 2021, Chapter 23) has the potential to accurately emulate LV mechanics. A GNN can act directly on the same computational mesh representation of the LV geometry as the forward simulator, without requiring a low-order approximation. This is in contrast with existing work on emulation of the LV displacement field, which makes low-order approximations to the displacement field (Buoso et al. 2021) or LV geometry (Maso Talou et al. 2020). However, designing a GNN architecture that can obtain high accuracy on this challenging emulation problem, while simultaneously delivering large computational savings over the numerical forward simulator, is a substantial research project in its own right. It is hence beyond the scope of this study, but will be the subject of future work.

One limitation of the the SA experiments presented in this study was the use of uninformative prior distributions for the input parameters of the forward model. Uninformative priors were used as the true population distribution is unknown. An alternative approach would be to *construct* a prior distribution via rejection sampling, which has the potential to better approximate the population distribution. This can be done by first sampling an input point from the uniform distribution over the input space, before running a simulation from that point. If the resulting displacement field exhibits non-realistic behaviour, the sample is rejected. Several criteria can be used to specify non-realistic behaviour. One possible criterion could be that the magnitude of the inflation of the left ventricle from start to end diastole does not exceed some prescribed value, which is assumed to be the limit of what is likely to be observed in experimental data. Repeated samples obtained with this procedure can better reflect samples from the true population distribution than those from the uniform and log-uniform priors. We did not pursue this idea in this work as this procedure is likely to produce a prior distribution relatively similar to the uniform distribution (albeit at higher computational costs), since we have observed that most extreme behaviour of the simulated volume and strain values occurs for very low material stiffness parameter values, where the uniform prior already assigns low probability mass. We have already seen broad consistency between the SA results under the uniform and log-uniform priors, despite the large differences in how these distributions assign probability mass to the material parameter space.

In the I-UQ study, the noise added to the circumferential strains was assumed to be iid Gaussian distributed, allowing us to focus on I-UQ without the confounding effect of measurement bias. The effect that measurement bias has on inference is similar to that of model discrepancy, resulting in parameter estimates that have less interpretability in terms of physical descriptions (Lei et al. 2020). As such, the methods proposed by Kennedy and O’Hagan (2001) for accounting for model discrepancy could similarly be applied in this context. However, Brynjarsdóttir and O’Hagan (2014) showed the importance of including prior knowledge in such a model, making this a fairly substantial direction of future research, requiring first a consideration of the limitations of the model and measurement process (this could be based on studies carried out in the literature, such as Berberoğlu et al. (2021)), and then a way for including this knowledge in the model. The first author has started preliminary work to improve the model in this direction, which can be found in Lazarus (2022, Chapter 9).

The quantities of interest are chosen based on routinely available in vivo CMR measurements. For example, $\text {LVV}$ and circumferential strains have been widely used for clinical diagnosis. Future studies shall extend the model to consider both passive filling and active contraction (Campos et al. 2020), and to include other measurements such as ejection fraction, the rate of systolic pressure increase, etc., and further extend it to a coupled electro-mechanics full-heart model (Levrero-Florencio et al. 2020).

We have used a well-established model for left ventricle passive mechanics, instead of a more complex model. This is because the purpose of the study was on sensitivity analysis and uncertainty quantification of passive mechanics, with emphasis on experiments that are feasible to perform using data available in-clinic and that can provide insights into cardiac function. We also employed a widely used strain invariant-based H-O model for the myocardium (Gao et al. 2017a; Peirlinck et al. 2019). However, the existing literature has not comprehensively investigated the practical parameter identifiability of the H-O model given data available from non-invasive measurements. We expect that the statistical approaches developed in this study can be readily applied to other myocardial strain energy functions used in Genet et al. (2014) and Hadjicharalambous et al. (2017).

A further future research direction is to address the fact that geometrical uncertainty has not been considered yet, which can arise from the imaging protocol, the segmentation procedure and the reconstruction, etc. For a detailed review of uncertainties associated with cardiac models, the reader is referred to Mirams et al. (2016) and the presentations given at the workshops of “The Fickle heart” programme^1^ held at the Isaac Newton Institute, 2019 (Mirams et al. 2020). Finally, we would like to mention that only the data at end-diastole has been used for parameter inference, while the SA and I-UQ analysis in this study has suggested that extra measurements at high pressure will improve parameter identifiability. One potential way could be the combination of ex vivo volume-pressure relationship from the Klotz curve (Klotz et al. 2006) and in vivo measurements at end-diastole.

**Table 7:** Gaussian process learned hyper-parameter values

Output	$\lambda _{a}$	$\lambda _{b}$	$\lambda _{a_{\mathrm {f}}}$	$\lambda _{b_{\mathrm {f}}}$	$\lambda _{a_{\mathrm {s}}}$	$\lambda _{b_{\mathrm {s}}}$	$\lambda _{a_{\mathrm {fs}}}$	$\lambda _{b_{\mathrm {fs}}}$	$\lambda _{\mathrm {EDP}}$	$\lambda _{\alpha _{\mathrm {endo}}}$	$\lambda _{\alpha _{\mathrm {epi}}}$	$\sigma _{f}$	$\eta$
LVV	1.8	0.8	4.1	1.6	6.8	67.2	41.2	118.5	27.2	6.5	6.21	11.2	0.06
$\varepsilon _\text {cc}$	1.2	1.7	2.2	2.7	289.9	653.7	22.5	645.3	2.1	2.7	3.0	1.0	0.05
$\varepsilon _\text {ll}$	0.9	1.1	2.3	2.8	$1.4\times 10^{5}$	$1.4\times 10^{5}$	45.2	74.0	2.1	3.1	3.0	4.4	0.06
$\varepsilon _\text {rr}$	1.1	0.9	2.8	1.6	3.4	58.9	2.8	19.9	24.0	2.7	8.6	4.4	0.06

## Conclusion

This paper has provided a comprehensive sensitivity analysis of the H-O model, proposing a new parameterization that has been studied using I-UQ. This involved the use of Bayesian inference with a likelihood function approximated using a statistical emulator. To our knowledge, the final parameterization is different from any previously proposed in the literature and, unlike the existing models, it comes with a quantitative justification from a sensitivity analysis. The results of the I-UQ study were found to be consistent with those of the SA. In particular, we observed good identifiability of *a* and $a_{\mathrm{f}}$ in the in vivo pressure range. These parameters are associated with the toe region of the myocardial mechanical response. We also found that *b* and $b_{\mathrm{f}}$ cannot be reliably inferred without the inclusion of high pressure data. This should motivate future work on the inclusion of high-stretch behaviour of the tissue in the inference framework, perhaps from ex vivo studies like the Klotz curve (Klotz et al. 2006). For these methods to be reliable in a clinical setting, this must all be done within a proper statistical inference framework, taking account of the uncertainties introduced by the data and the model.

## Figures and Tables

**Fig. 17:**
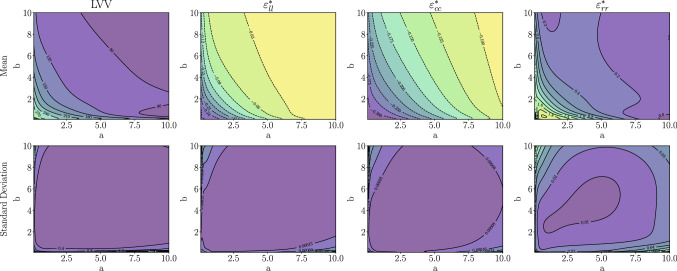
Gaussian process contour plots. The first row shows the posterior mean, and the second the posterior variance of the GP emulators, over $[a,b] = [0.1, 10]^2$. Each column corresponds to one of the four QoIs considered in the SA experiments. Other input parameters are fixed as follows: $a_{\mathrm {f}}=1$, $b_{\mathrm {f}}=1$, $a_{\mathrm {s}}=0.69$, $b_{\mathrm {s}}=1.11$, $a_{\mathrm {fs}}=0.31$, $b_{\mathrm {fs}}=2.58$, $\text {EDP} = 10$ mmHg, $\alpha _{\mathrm {endo}}=60^{\circ }$, $\alpha _{\mathrm {epi}}=-90^{\circ }$

**Fig. 18:**
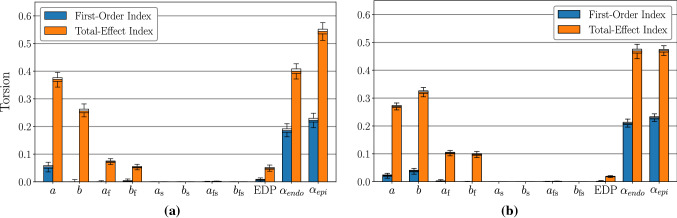
SA1 results for the apical torsion-**a** is for uniform prior, and **b** for log-uniform

## Data Availability

The datasets supporting this article have been uploaded to Github as part of the electronic supplementary material, https://github.com/HaoGao/ho-uncertainty-quantification.

## Appendix

### A: Additional emulator validation results

The values of the inferred kernel hyper-parameter values from Equation (15) for the Gaussian process emulators used to perform SA1 are given in Table 7. We can see that the input parameters that were found to have high sensitivity, such as *a* and *b*, tend to have low $\lambda$ values. On the other hand, inputs such as $a_{\mathrm {fs}}$ and $b_{\mathrm {fs}}$ which had lower sensitivity, tend to have higher $\lambda$ values, indicating that the function changes more slowly with respect to these inputs. In addition, we have produced in Fig. 17 plots of the posterior mean and standard deviation of the emulators over [*a*, *b*] space, with all other inputs held fixed. In each case, the mean value is largest in magnitude for smaller values of *a* and *b*, while the standard deviation is more stable across the input space.

### B: Sensitivity analysis of the apical torsion

Figure 18 shows the apical torsion as alternative QoI for SA1. The apical torsion is defined as the twist angle of the apex at the current configuration (i.e. at end-diastole) compared to the reference state. The general trend of the Sobol indices of the material parameters for the apical torsion is similar to the indices obtained from the other four QoIs considered in Section 3.4.2, see Fig. 5. In particular, the main pattern is confirmed, namely, that parameters $a_\text {s}, b_\text {s}, a_\text {fs}$ and $b_\text {fs}$ have effectively zero sensitivity indices and can therefore be excluded from the subsequent inference step. For the fibre generation parameters $\alpha _\text {endo}$ and $\alpha _\text {epi}$, the observed indices are higher compared to the values observed for the four output QoIs displayed in Fig. 3.4.2.

### C: Change in I-IQR over parameter space

Plots of changing I-IQR over parameter space, which are produced using the procedure in Table 8, are shown in Fig. 19. For all four parameters, the identifiability decreases significantly as we move from the description of a soft material (smaller material parameters) to a stiffer material (larger material parameters). Where the material is stiff, the parameters are less identifiable and the effect that pressure has on the parameter identifiability cannot be observed. Contrastingly, where the material is soft, we are able to see the effect that pressure has on the parameter identifiability. Overall, this means we have low variation in identifiability at pressures where a particular parameter is non-identifiable but more variation in identifiability of a given parameter at pressures where the parameter identifiability improves.

**Table 8:** Algorithm for producing the contour plots of Fig. 19

Producing a typical I-IQR contour plot.
1. Generate grid of $a-b$ values in the range 0.5-9.5.
2. For each point in the grid, generate synthetic data by evaluating the emulator, with $a_{\mathrm{f}}$ and $b_{\mathrm{f}}$ fixed equal to 1.
3. Add *N*(0, 0.1) noise to the normalized test data (normalized by the mean and standard deviation of the emulator training data).
4. For each test point, obtain samples of the material parameters using HMC and use these to approximate the IQR of the posterior distribution.
5. Create contour plot using the I-IQR at each point on the grid of material parameter values.

## Footnotes

1 https://www.newton.ac.uk/event/fht.
